# *APOE* ɛ4 and Insulin Resistance Influence Path-Integration-Based Navigation through Distinct Large-Scale Network Mechanisms

**DOI:** 10.14336/AD.2024.0975

**Published:** 2025-11-18

**Authors:** Karel M. Lopez-Vilaret, Marina Fernandez-Alvarez, Anne Bierbrauer, Nikolai Axmacher, Jose L. Cantero, Mercedes Atienza

**Affiliations:** ^1^Laboratory of Functional Neuroscience, Pablo de Olavide University, Seville, Spain.; ^2^CIBER de Enfermedades Neurodegenerativas (CIBERNED), Instituto de Salud Carlos III, Madrid, Spain.; ^3^Institute for Systems Neuroscience, Hamburg, Germany.; ^4^Department of Neuropsychology, Institute of Cognitive Neuroscience, Faculty of Psychology, Ruhr University Bochum, Universitätsstraße 150, Bochum, Germany.

**Keywords:** aging, insulin resistance, *APOE* ɛ4;, network segregation, path integration

## Abstract

Path integration (PI), which supports navigation without external spatial cues, is facilitated by grid cells in the entorhinal cortex. These cells are often impaired in individuals at risk for Alzheimer’s disease (AD). However, other brain systems can compensate for this impairment, especially when spatial cues are available. From a graph-theoretical perspective, this compensatory mechanism might manifest through changes in network segregation, indicating shifts in distinct functional roles among specialized brain regions. This study explored whether similar compensatory mechanisms are active in *APOE* ε4 carriers and individuals with elevated insulin resistance, both susceptible to entorhinal cortex dysfunction. We applied a graph-theoretical segregation index to resting-state fMRI data from two cohorts (aged 50-75) to assess PI performance across virtual environments. Although insulin resistance did not directly impair PI performance, individuals with higher insulin resistance demonstrated better PI with less segregated brain networks, regardless of spatial cue availability. In contrast, the *APOE* effect was cue-dependent: ε4 heterozygotes outperformed ε3 homozygotes in the presence of local landmarks, linked to increased sensorimotor network segregation. When spatial cues were absent, ε4 carriers exhibited reduced PI performance due to lower segregation in the secondary visual network. Controlling cortical thickness and intracortical myelin variability mitigated these *APOE* effects on PI, with no similar adjustment made for insulin resistance. Our findings suggest that ε4 carriers depend on cortical integrity and spatial landmarks for successful navigation, while insulin-resistant individuals may rely on less efficient neural mechanisms for processing PI. These results highlight the importance of targeting insulin resistance to prevent cognitive decline, particularly in aging navigation and spatial cognition.

## INTRODUCTION

The pathology of Alzheimer's disease (AD) begins decades before the onset of symptoms. While research has historically considered episodic memory deficits as an initial sign, increasing evidence suggests that spatial disorientation may represent an even earlier manifestation of the disease [1]. Substantiating this idea, a study in asymptomatic middle-aged adults has identified challenges in path integration (PI)—the ability to estimate self-location through tracking distances and directions from a reference point—as a specific predictor of AD risk [2]. The entorhinal cortex, one of the initial sites affected by tau pathology in AD [3], also constitutes a pivotal region responsible for PI processes [4]. In the absence of spatial cues, these processes are predominantly facilitated by grid cells in the medial entorhinal cortex [2,5-9]. Conversely, when these cues are available, supplementary neural systems located in the hippocampus [10] and retrosplenial cortex [6,11] may be engaged.

PI deficits have also been linked to the apolipoprotein ɛ4 isoform (*APOE* ɛ4), the most significant genetic risk factor for sporadic AD [2,6,12-14]. This association appears to be influenced by age and gender [2,15]. Indeed, carriers of the ɛ4 allele may perform better or worse in PI tasks compared to non-carriers, depending on whether they are women or men, respectively, an effect that is particularly evident in younger individuals [15]. In older adults, PI deficits associated with the presence of the *APOE* ɛ4 genotype manifest when individuals cannot rely on additional spatial cues that are processed by compensatory brain networks [2,6].

The neurobiological mechanism(s) through which *APOE* ɛ4 influences PI remains elusive. The ɛ4 isoform increases AD risk via a number of different biological pathways including (among others) failures in cholesterol metabolism, amyloid clearance, insulin-dependent energetic metabolism, and the regulation of glucose homeostasis [16]. Indeed, accumulating research suggests that cognitive decline and the development of AD pathology is often preceded by brain insulin resistance [17], as revealed by diminished or even absent brain response to insulin. Accordingly, insulin resistance disorders such as obesity, type 2 diabetes, and metabolic syndrome are closely linked to AD [18], which, in turn, is characterized by impaired insulin signaling in the brain and decreased levels of insulin and insulin receptors [19,20].

Although insulin resistance can manifest independently in the brain and the periphery, much evidence supports that one can also lead to the other [21]. In this regard, peripheral insulin resistance has been reported subsequent to central insulin dysregulation in a mouse model of AD [22]. Likewise, another study found that peripheral insulin resistance caused by long-term western-diet feeding was followed by disrupted insulin signaling and by the development of AD pathology in the mouse entorhinal cortex [23]. Interestingly, a study in human *APOE* ɛ4-targeted replacement mice has shown that both aging and peripheral insulin resistance induce impairment of brain insulin signaling [24]. Considering that brain response to insulin can be compromised by peripheral insulin resistance [23,25,26], but also by the *APOE* ɛ4 genotype in an age-dependent manner [24], and that the entorhinal cortex is especially vulnerable to impairments of brain insulin signaling [23], we posit that *APOE* ε4 carriers and individuals with elevated peripheral insulin resistance are likely to encounter greater challenges in estimating PI when spatial cues are lacking. Even though PI processes are particularly supported by entorhinal grid cells, the variability in accuracy is largely explained by the specific patterns of interaction among sensorimotor, control, spatial attention, and spatial working memory systems [27,28]. It is likely, therefore, that the genetic predispositions and physiological abnormalities that hinder the execution of PI processes require compensatory involvement from brain regions capable of integrating idiothetic and allocentric information, such as the retrosplenial cortex [6,11]. However, this integrative communication capacity may be compromised by aberrant central insulin signaling, which has been shown to be responsible for neurovascular uncoupling through astrocytes in aging [29]. Therefore, we postulate that both *APOE* ɛ4 carriers and individuals with high peripheral insulin resistance will need enhanced communication between large-scale brain networks, especially associative networks, to accurately estimate PI. However, we do not rule out that spatial task performance in these vulnerable individuals may also depend on the segregation of sensorimotor networks, particularly those involved in visual processing. This information has been shown to be crucial when other brain modules, such as the vestibular system, are impaired [30,31]. This is particularly relevant to the present study, as the virtual nature of the spatial task does not provide input to the vestibular system. To evaluate this hypothesis, we employed the segregation index, which assesses the degree of functional specialization of brain networks by measuring the strength of functional connectivity (FC) within individual resting-state networks relative to their connectivity with other brain networks [32]. This measure is particularly pertinent to understanding PI, as it captures the efficiency of information processing and integration across these networks, reflecting the brain's ability to navigate spatial environments effectively.

Given the evidence linking the *APOE* ɛ4 to cortical thinning throughout life [33] and reduced intracortical myelin in older adults [34], alongside the established impact of cortical thickness [35] and intracortical myelin content on FC [36,37], we hypothesize that variability in cortical thickness and intracortical myelin may further influence the moderating role of brain system segregation on the relationship between *APOE* ε4 genotype and PI. Finally, as insulin resistance has been associated with progressive atrophy in cortical regions affected by early AD in asymptomatic, late middle-aged individuals [38], the above-mentioned hypothesis can be extended to encompass the moderating role of insulin resistance.

## MATERIALS AND METHODS

### Participants

Two cohorts of cognitively unimpaired late middle-aged and older adults participated in the study: Cohort I (N=53; 63.1 ± 5.7 years; range: 54-75 years; 26 females) and Cohort II (N=49; range: 52-75 years; 28 females). Participants were recruited from senior citizen’s associations, health-screening programs, and hospital outpatient services. This study employs a retrospective design, drawing on previously collected data initially gathered for different research purposes. Behavioral results from Cohort I have been reported in a previous study assessing the effects of the *APOE* genotype on PI estimation [6], while Cohort II was specifically recruited to evaluate outcomes from a shorter version of the Apple Game (unpublished data). All participants met the following criteria: i) normal global cognitive status as revealed by scores ≥ 25 in the Mini-Mental State Examination; ii) global score of 0 (no dementia) in the Clinical Dementia Rating; iii) functional independence as assessed by the Spanish version of the Interview for Deterioration in Daily Living Activities [39]; iv) scores ≤ 5 (no depression) in the short form of the Geriatric Depression Scale [40]; v) not be taking medications that affect cognition; and vi) not being a carrier of the ɛ2 allele or homozygous for the ɛ4 allele. All participants gave informed consent to the experimental protocol approved by the Ethical Committee for Clinical Research of the Junta de Andalucía according to the principles outlined in the Declaration of Helsinki.

### Virtual spatial navigation task

The cohort I and II performed a long and a short version, respectively, of the spatial task known as the Apple Game. From now on we will refer to these two samples depending on whether they have performed the long or the short version of the Apple Game as S_longAG_ and S_shortAG_, respectively. The long version, illustrated in Supplementary Figure 1, is described in detail in [6]. Briefly, participants navigated in different virtual environments using a joystick (GXT 555 Predator, Trust, Netherland) that allowed them to move in the virtual environment. Each trial consisted of 4 phases: i) picking up a basket; ii) passing through 0-4 distractors (0-4 trees without apples); iii) passing through a tree with a red apple; and iv) returning to the place where the basket was initially located. Once participants reached the original location of the basket, they pressed a button and received zero to three stars, depending on the Euclidean distance between their estimated location and the correct location of the basket (drop error). The smaller the drop error, the better the PI estimate.

PI was assessed in three virtual environments that differed in terms of the presence/absence of supporting spatial cues. In the Pure PI (PPI) condition, the environment contained no spatial cues. It consisted of an infinite grass plane with a blue sky rendered at infinity. In the Boundary PI (BPI) condition, a circular stone wall surrounded the same environment; and in the landmark PI (LPI) condition, a lighthouse served as a proximal landmark in that environment.

Participants of the S_shortAG_ navigated a virtual environment that incorporated mountains rendered at infinity. The introduction of distal landmarks was motivated by the difficulty shown by the S_longAG_ participants to perform this task successfully even in the simplest difficulty condition. In addition to introducing distal landmarks to facilitate orientation, we reduced the number of distractor trees to one. The variability in difficulty across trials was linked to the distance the participant had to travel to get from the basket location to the apple tree (i.e., outgoing distance) and the distance from the apple tree to the basket location (i.e., incoming distance). Finally, in order to shorten the time requirements, we decided to remove the BPI condition from the experimental protocol. Therefore, the short version of the Apple Game task included a total of 24 trials compared to 96 trials in the long version.

### Anthropometric, cardiovascular and biochemical measurements

Fasting blood samples were collected at 8:00-10:00 AM in all participants, immediately processed, and stored at -80°C until analysis. Serum levels of glucose, triglycerides, low-density lipoprotein (LDL) and high-density lipoprotein (HDL) cholesterol were obtained with the automated A15 Random Access Analyzer® (Biosystems, Spain) using Biosystems reagents. Serum insulin levels were determined with Quantikine ELISA kits (R&D Systems, Minneapolis, MN, USA) following the manufacturer’s instructions. Insulin determinations were run in duplicates, and the average of the two measurements was used for statistical analysis. Replicas that differed more than 20% were repeated.

The body mass index (BMI) was calculated by dividing weight in kilograms by square height in meters. Waist circumference was used as a measure of central obesity. Measurements of systolic/diastolic blood pressure were obtained in sitting position, after spending 10 min in a quiet room maintained at a constant temperature of 22ᴼC.

Insulin resistance was assessed through the Homeostatic Model Assessment of insulin resistance (HOMA-IR). The HOMA-IR score was obtained by dividing the product of fasting serum insulin (mU/l) and glucose (mmol/l) by 22.5 [41]. SI units for insulin were transformed into conventional units by dividing by a factor of 6 [42].

As insulin resistance is closely linked to cardiovascular factors comprising metabolic syndrome, we opted to include this information in the various models as a potential confounding factor. To avoid the loss of information due to the dichotomous definition of metabolic syndrome, we computed the continuous metabolic syndrome score (MtbS) proposed in a previous study [43], which provides a measure comparable across different studies and populations. The higher the MtbS score, the worse the metabolic status of the participant. The MtbS was computed by applying the following equation:

$$\mathrm{MtbS}=2\times \frac{\mathrm{Waist}}{\mathrm{Height}}+\frac{\mathrm{Glucose}}{5.6\mathrm{mmol}/l}+\frac{\mathrm{Triglycerides}}{1.7\mathrm{mmol}/l}+\frac{\mathrm{Systolic pressure}}{130\mathrm{mmHg}}-\frac{\mathrm{HDL}}{40\left(M\right)\mathrm{or}50(F)}(1)$$

### APOE genotyping

Genomic DNA was isolated from blood using a standard salting-out protocol [44], and *APOE* polymorphisms were determined with pre-designed TaqMan® single nucleotide polymorphism genotyping assays (assay ID: C_3084793_20 for rs429358 and assay ID: C_904973_10 for rs7412; Applied Biosystem™, Thermo Fisher Scientific).

### Structural and functional MRI data acquisition

MRI brain images were acquired in a 3T Philips Ingenia CX MRI scanner equipped with a 32-channel receiver head coil (Philips, Best, Netherlands). Prior to scanning, participants were screened for contraindications to MRI, and detailed instructions were provided to ensure they understood the procedure. Head motion was minimized by placing foam padding around the subject's head. The following MRI sequences were acquired in the same session: i) 3D T1-weighted (T1w) Magnetization-Prepared Rapid Gradient Echo (MP-RAGE) sequence in the sagittal plane: repetition time (TR)/echo time (TE)-=-2600-ms/4.7-ms, flip angle (FA)-=-90°, acquisition matrix-=-384-×-384 mm, voxel resolution in acquisition =-0.65-mm^3^ isotropic, resulting in 282 slices without gap between adjacent slices; ii) 3D T2w VISTA Turbo Spin Echo sequence in the sagittal plane: TR/TE: 2500 ms/251 ms, FA = 90°, acquisition matrix = 384 x 384 mm, voxel resolution in acquisition-=-0.65-mm^3^ isotropic, resulting in 282 slices without gap between adjacent slices; and iii) T2w Fast Field Echo images using a blood-oxygen-level-dependent (BOLD) sensitive single-shot Echo-Planar imaging (EPI) sequence in the axial plane: TR/TE: 2000 ms/30 ms, FA = 80°, acquisition matrix = 80 x 80 mm, voxel resolution in acquisition = 3 mm^3^ isotropic, resulting in 35 slices acquired in posterior to anterior phase-encoding direction with 1 mm of gap between adjacent slices. Pulse and respiratory rates were simultaneously recorded using the scanner’s built-in pulse oximeter placed on the left-hand index finger and a pneumatic respiratory belt strapped around the upper abdomen, respectively. Before starting the acquisition of the EPI sequence, participants were asked to remain still and keep their eyes closed without falling asleep. We acquired 250 EPI scans preceded by 4 dummy volumes to allow time for equilibrium in the spin excitation. To facilitate optimal B1 shimming, a B1 calibration scan was applied before starting the EPI sequence. Brain images were visually examined after each MRI sequence; they were repeated if evident artifacts were identified. All participants underwent the same protocol in the same scanner at the research MRI facility located at Pablo de Olavide University.

### MRI preprocessing, cortical thickness estimation, and T1w/T2w ratio map generation

Structural MRI data was processed using the pipeline of Freesurfer v6.0 (https://surfer.nmr.mgh.harvard.edu/). The Freesurfer’s pipeline included brain extraction, automated tissue segmentation, generation of white matter (WM) and pial surfaces, correction of surface topology and inflation, co-registration, and projection of cortical surfaces to a sphere for the purpose of establishing a surface-based coordinate system [45]. Pial surface misplacements and erroneous WM segmentation were manually corrected on a slice-by-slice basis by experienced technicians. Cortical thickness was defined as the average shortest distance measured from the pial surface to the gray matter-white matter (GM-WM) boundary at each vertex across the cortical mantle.

T2w images were registered to T1w images with *bbregister* using a trilinear interpolation method and a boundary-based cost function constrained to 6 degrees of freedom [46]. In order to correct intensity inhomogeneities, the *N4BiasFieldCorrection* function was applied to individual T1w and T2w images separately [47]. We then computed individual bias field corrected-T1w/T2w ratio volumes, which were sampled halfway between the WM and GM surface. To mitigate contamination of cortical GM intensity by intensities of WM and cerebrospinal fluid (CSF), the tissue fraction effect was corrected in individual T1w/T2w ratio maps using the geometric transfer matrix-derived region-based voxel-wise method implemented in PETsurfer [48].

The cortical thickness and T1w/T2w ratio map of each subject was projected onto the average subject’s cortical surface using 7th order (i.e., 163842 vertices) and 6th order (i.e., 40960 vertices) icosahedron tessellations. Cortical surfaces obtained with the 7th order tessellation were used to determine differences in GM integrity between the two samples and to assess the impact of the *APOE* genotype and insulin resistance on GM integrity. As the 6th order tessellation is the one that most closely approximates the surface spaces employed to generate the parcellation of the cerebral cortex [49], it was used to eliminate the contribution of GM integrity from FC before recomputing network segregation (for more detail, see the subsection Network segregation partialized by GM integrity). For statistical analyses on the 7th order icosahedron tessellation, we applied non-linear spherical wavelet-based de-noising schemes [50], whereas for parcellation-based analysis, we applied a small Gaussian kernel with full width at half maximum (FWHM) of 2 mm. All processing steps were visually checked for quality assurance.

Preprocessing of resting-state fMRI was done in the subject-space using functions of the AFNI software (version 20.3.03). It involved (i) elimination of high-frequency spikes; (ii) application of RETROICOR to minimize time-locked cardiac and respiratory motion artifacts on cerebral BOLD signals; (iii) correction of time differences in slice-acquisition; (iv) EPI scan alignment using rigid body motion correction with the first volume as reference; (v) correction of field distortions based on fMRI sequence acquired with inverted phase-encoding direction; (vi) co-registration of aligned EPI scans to their corresponding T1w volumes; (vii) nuisance regression to remove linear drifts and minimize the impact of non-neuronal fluctuations by including six head motion parameters with their first-order derivatives, time series of mean total WM/CSF signal intensity, and pulse/respiratory fluctuations plus their derivatives; (viii) band-pass filtering to 0.01-0.1 Hz; and (ix) nonlinear transformation from individual functional space into MNI152 space.

Before proceeding with detailed analyses, we conducted quality checks on fMRI data to identify and mitigate potential artifacts and confounding factors that could distort the data. Specifically, we detected motion-related outliers using the AFNI functions *enorm* and *3dToutcount*, removing volumes where more than 5% of voxels exceeded the median absolute deviation of the time series or when head movement exceeded 0.3 mm. We also verified the proper registration of EPI to T1 by superimposing volumes or masks using neuroimaging software such as MRIcron or AFNI for visual confirmation. Additionally, we ensured that physiological variables were correctly acquired by confirming that pulse and respiration data were properly aligned with brain volumes and that no parts of the recordings were missing. Finally, we visually inspected the EPI signal after applying RETROICOR to confirm the correction of physiological artifacts, looking for noise and variance reduction indicated by brighter voxels, particularly around the brain ventricles. Well-differentiated ventricular areas suggested successful correction, while poor differentiation led to the classification of the subject as an outlier due to insufficient artifact correction.

### Computation of network segregation

We applied the segregation index proposed by Chan et al. [32]. This index considers the differences in mean within-network FC and mean between-network FC as a proportion of mean within-network FC. The index is represented in the following equation:

$$\mathrm{Network segregation}=\frac{{\overset{-}{Z}}_{W}-{\overset{-}{Z}}_{B}}{{\overset{-}{Z}}_{W}}(2)$$

where ${\overset{-}{Z}}_{W}$is the mean Fisher *z*-transformed *r* between nodes within the same network (within-network FC) and ${\overset{-}{Z}}_{B}$is the mean Fisher *z*-transformed *r* between nodes of one network to all nodes in other networks (between-network FC). In order to define the nodes, we used two different surface-based parcellation schemes, both derived from resting-state FC-boundary maps [49,51]. The Gordon parcellation scheme [49], illustrated in Supplementary Figure 2 (left panel), includes 333 highly functionally homogeneous parcels (nodes) that not only overlap with known architectonic areas, but they also have a network structure similar to the one obtained when every voxel in the brain was used as a node [52]. This brain system organization included 4 sensorimotor networks, particularly the auditory (AN), visual (VN), hand somatomotor (hSMN), and mouth SMN (mSMN), and 8 associative networks such as the default mode (DMN), frontalparietal (FPN), dorsal attention (DAN), ventral attention (VAN), salience (SN), cingulo-opercular (CON), cingulo-parietal (CPN), and retrosplenial-temporal network (RTN). The number of parcels forming each network is shown in Supplementary Table 1. The segregation index for parcels in the orbitofrontal cortex and anterior ventral lateral temporal lobe (referenced as ‘none network’ in Supplementary Table 1) was not computed because they are likely to be noisy and unreliable due to low SNR [53]. The Ji parcellation scheme [51], illustrated in Supplementary Figure 2 (right panel) and Supplementary Table 1, does not include the RTN, but it includes methodological innovations that contribute to improve the precision of the partitions and to delineate new functional networks such as the posterior multimodal (PMN), ventral multimodal (VMN), orbito-affective (OAN) and language network (LN). Of these, the PMN, which consisted of bilateral dorsomedial parietal lobe, bilateral temporo-parietal-occipital junction and right dorsocaudal temporal lobe, is particularly interesting due to its role in spatial navigation [51].

Before computing the segregation index, we averaged the BOLD signal across vertices within each parcel, computed Pearson’s correlation across time between each pair of parcels (edges of the network), applied the Fisher *z*-transformation, and removed the negative *z*-values.

### Network segregation partialized by GM integrity

Next, we calculated two partialized segregation indices, one removing the contribution of cortical thickness and the other removing the contribution of intracortical myelin content derived from the T1w/T2w ratio. For this, we also averaged the cortical thickness and the T1w/T2w ratio across vertices within parcels. For each parcellation scheme, we first computed the partialized within-network FC and partialized between-network FC by fitting two regression models of the form:

$$y_{i=}=\beta_{0}+\beta_{1}R_{i}+e_{i}(3)$$

where yis the outcome variable (i.e., either $Z_{W}$ or $Z_{B}$), R refers to a continuous target predictor (i.e., either cortical thickness or T1w/T2w ratio), the βs are fixed unstandardized model parameters to be estimated, eis the residual, and i refers to the parcel within the whole cortical surface. We next obtained the partialized FC ( ${\mathrm{PFC}}_{i}$) between parcels within and between functional networks by computing the following equation:

$${\mathrm{PFC}}_{i}=\overset{n}{\underset{i=1}{\Sigma }}\left(y_{i}-\beta_{1}R_{i}\right)(4)$$

We next obtained the partialized within-network FC ( ${\overset{-}{Z}}_{W}$), and the partialized between-network FC ( ${\overset{-}{Z}}_{B}$), by averaging the z-transformed ${\mathrm{PFC}}_{i}$ across parcels for each functional network. Finally, we applied equation (2) to obtain the segregation index of each functional network partialized either by the cortical thickness or the T1w/T2w ratio.

### Statistical analysis

First, we applied the Box-Cox transformation to all positive dependent variables and the Yeo-Johnson transformation to dependent variables including positive and negative responses [54]. These transformations contribute to stabilizing the variance, improve normality and improve the accuracy of predictions derived from linear regression analysis. The independent continuous variables were Z transformed. The analyses involving network segregation as either dependent or independent variable were performed for sensorimotor and associative networks resulting from averaging the corresponding networks in each parcellation scheme, as well as for the different single networks.

We applied linear regression models (LM) and linear mixed-effect regression models (LME) by using the functions *fitlm* and *fitlme* implemented in MATLAB. To validate the assumptions, we assessed normality of residuals through the Shapiro-Wilk test and visual inspection of QQ plots and histograms. Homoscedasticity and independence were confirmed using residuals versus fitted values and residuals versus index plots. Potential outliers were identified through visual inspection of residuals and statistical methods, such as Cook's distance, and addressed to ensure they did not unduly influence the model. The Variance Inflation Factor (VIF) function was used to diagnose multicollinearity among predictor variables. VIF values exceeding 5 were considered indicative of problematic multicollinearity. This was the case for the models performed at the single network level, whereby we were forced to repeat the same model for each network separately. The type-I error resulting from multiple comparisons across networks was corrected by applying the Holm-Bonferroni correction of the family wise error rate (FWER) with the multiple testing toolbox implemented in MATLAB [55] (https://es.mathworks.com/matlabcentral/fileexchange/70604-multiple-testing-toolbox).

To control for potential confounding variables, we included age, sex, years of education, and BMI as covariates in the statistical models. Additionally, we accounted for comorbidities such as diabetes and cardiovascular issues through the continuous metabolic syndrome score (MtbS), which as mentioned above integrates risk factors including glucose levels, central obesity, dyslipidemia, and systolic blood pressure. The incoming distance was also included as covariate instead of the outgoing distance, as it has demonstrated a more pronounced effect on drop error [6]. Cognitive status was not included as a confounding variable due to differing neuropsychological tests across cohorts; however, all participants met specific inclusion criteria ensuring normal cognitive status.

In all cases where the variable of interest exerted a significant effect on the dependent variable, the local standardized effect size was estimated. For main effects, we computed Cohen's f^2^ to obtain the local effect [56], while in the case of interaction we computed δ (categorical × continuous) or ρ (continuous × continuous) by applying the approximation proposed by Bodner [57]. The interpretation of the effect size (i.e., small, moderate, or large) was denoted with superscript letters S, M, or L, respectively. The precision of effect sizes was estimated by computing the bias-corrected and accelerated (BCa) bootstrap 95% confidence intervals (CI_95%_) through the *bootci* function implemented in MATLAB.

For surface-based statistical analyses related to GM integrity, we used the SurfStat package (www.math.mcgill.ca/keith/surfstat/). Results were corrected for multiple comparisons using a hierarchical statistical model that first controls the FWER at the cluster level by applying random field theory over statistical maps smoothed with non-linear spherical wavelet-based de-noising schemes [50], and next controls the false discovery rate at the vertex level within each cluster over unsmoothed statistical maps [58]. Peaks of clusters that survived correction for multiple comparisons were used to establish the anatomical location of significant changes using the Desikan-Killiany atlas [59].

**Figure 1.**
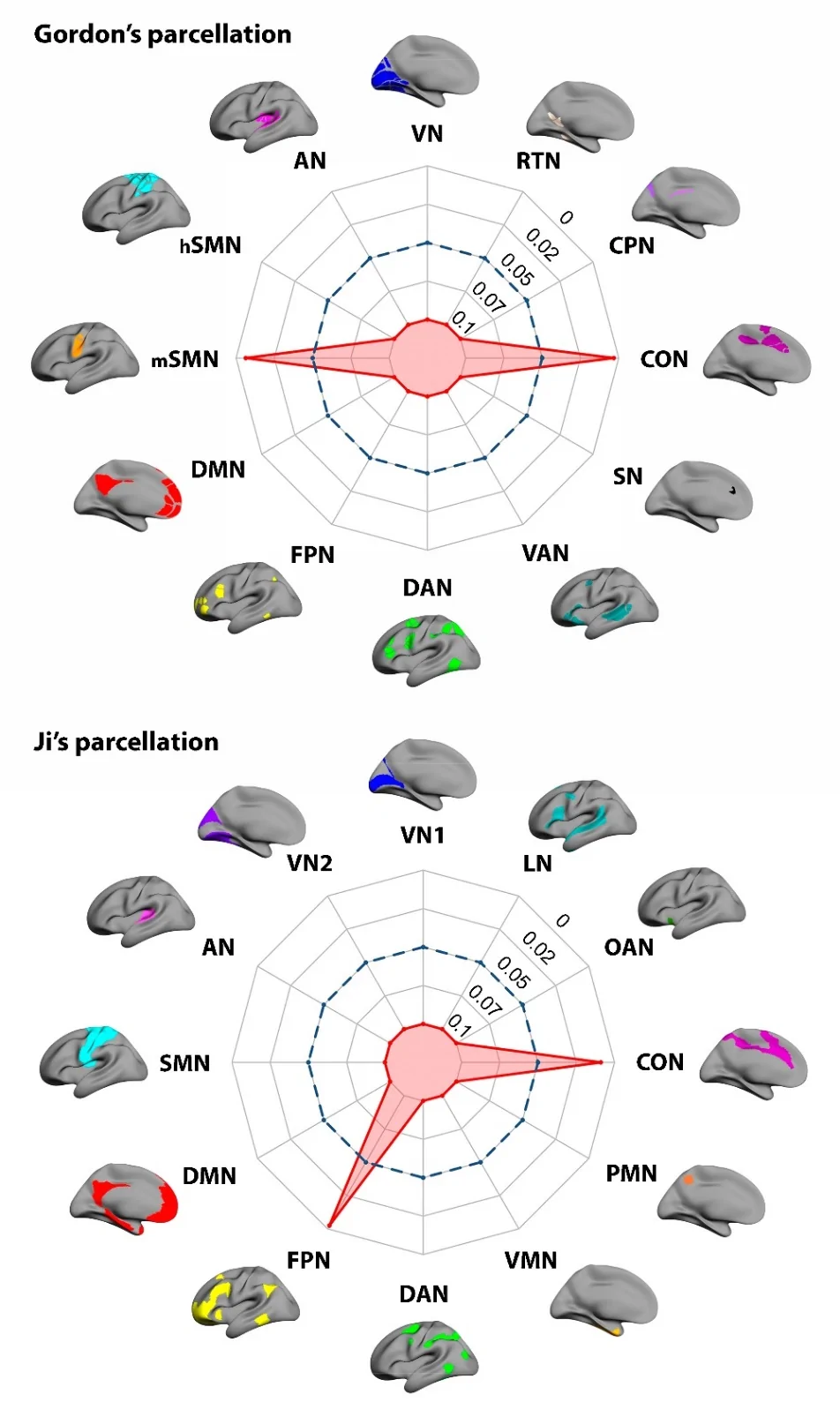
**Differences in functional network segregation between the two cohorts**. Significant differences between the two cohorts for each functional network in the Gordon and Ji parcellation scheme. The shaded blue line indicates the p value of 0.05. The solid red line indicates the p value of the LME model comparing network segregation between the S_longAG_ and S_shortAG_.

**Table 1:** Sample differences in the metabolic profile.

Metabolic parameter	S_longAG_ (N = 53)		S_shortAG_ (N=49)		p value	

	Mean ± SD	Nº individuals ≥ cut off	Mean ± SD	Nº individuals ≥ cut off	mean	cut off
**Insulin (mU/l)**	9.66 ± 6.12	—	7.54 ± 5.17	—	0.055	—
**HOMA-IR**	2.68 ± 1.89	—	1.73 ± 1.24	—	0.003	—
**MtbS score**	3.15 ± 0.59	—	2.19 ± 0.99	—	10^-7^	—
**BMI**	27.68 ± 4.08	25 overweight 13 obese	27.89 ± 4.79	23 overweight 14 obese	0.8	0.3
**Waist circumference (cm)**	90.84 ± 12.27	26	96.09 ± 12.65	29	0.014	0.019
**Systolic pressure (mmHg)**	122.77 ± 16.92	22	131.77 ± 25.86	29	0.029	0.08
**Glucose (mmol/l)**	6.10 ± 0.87	38	5.11 ± 1.45	8	10^-4^	0.5
**Triglycerides (mmol/l)**	1.19 ± 0.44	9	1.03 ± 0.84	4	0.2	0.9
**HDL cholesterol (mmol/l)**	1.59 ± 0.40	1	1.82 ± 0.50	0	0.004	1

Note: cut off 25 for overweight, 30 for obese; 94.5 for men and 89.5 for women for waist circumference (Martínez-Larrad et al., 2011); 130 for systolic pressure; 5.6 for glucose; 1.7 for triglycerides; 0.9 for men and 1.1 for women for HDL. p value after adjustment by age, education years, gender and *APOE*4.

## RESULTS

### Sample differences in the metabolic profile, network segregation, and GM integrity

We started by assessing sample differences in the metabolic profile. Table 1 includes the mean and standard deviation (SD) of HOMA-IR and cardiometabolic variables related to insulin resistance. It also shows the number of individuals who exceeded the cut-off point established in the dichotomous definition of metabolic syndrome for each of these variables [60]. The S_longAG_ showed a worse metabolic profile than the S_shortAG_ after adjusting the LM models by age, education years, gender and *APOE* genotype. Differences affected all evaluated metabolic parameters except for insulin, BMI and triglycerides. Considering the cut-off points, only waist circumference and systolic pressure differed between the two samples, with more people showing abnormal values in the S_shortAG_ than in the S_longAG_.

**Table 2:** Sample differences in the GM integrity.

*GM measurement* Contrast Brain cortical region (p_cluster_)	MNI	R^2^	F_9,95_	Size effect f^2^	CI_95%_
***Cortical thickness*** **S_longAG_ > S_shortAG_**					
**L entorhinal cortex (p = 0.0001)**	-22 3 -36	0.12	13.9	0.15^S^	0.03 - 0.36
**L lateral orbitofrontal cortex (p = 0.03)**	-15 28 -24	0.11	11.8	0.12^S^	0.02 - 0.30
**R superior temporal gyrus (p = 0.00001)**	49 1 -19	0.18	22.1	0.23^M^	0.08 - 0.46
**S_longAG_ < S_shortAG_**					
**L precentral gyrus (p = 0.00001)**	-28 -18 70	0.18	22.0	0.23^M^	0.08 - 0.46
**L fusiform gyrus (p = 0.0001)**	-25 -57 -13	0.14	16.5	0.17^M^	0.04 - 0.40
**L laterooccipital cortex (p = 0.02)**	-41 -87 -1	0.15	17.0	0.18^M^	0.05 - 0.40
**R precentral gyrus (p = 0.00001)**	28 -17 70	0.20	24.8	0.26^M^	0.09 - 0.53
***Intracortical myelin (T1w/T2w)***					
**S_longAG_ > S_shortAG_**					
**L lingual gyrus (p = 0.00001)**	-20 -78 -7	0.11	12.5	0.13^S^	0.03 - 0.31
**L lateral orbitofrontal cortex (p = 0.00001)**	-13 16 -24	0.12	14.1	0.15^S^	0.04 - 0.31
**L entorhinal cortex (p = 0.00001)**	-24 -3 -27	0.12	13.5	0.14^S^	0.02 - 0.37
**R temporal pole (p = 0.00003)**	-27 17 -36	0.13	15.3	0.16^M^	0.04 - 0.34
**R insula (p = 0.01)**	35 4 9	0.07	7.2	0.07^S^	0.01 - 0.21
**R superior parietal cortex (p = 0.02)**	-10 -74 44	0.06	6.7	0.07^S^	0.01 - 0.19
**R isthmus cingulate gyrus (p = 0.00003)**	8 -40 25	0.10	11.0	0.11^S^	0.02 - 0.27
**R precentral gyrus (p = 0.00003)**	53 2 42	0.09	10.4	0.11^S^	0.01 - 0.28
**R lingual gyrus (p = 0.0001)**	8 -58 6	0.10	10.8	0.11^S^	0.02 - 0.27
**R lateral orbitofrontal cortex (p = 0.0003)**	16 15 -16	0.11	12.7	0.13^S^	0.03 - 0.30
**R superior temporal gyrus (p = 0.0005)**	66 -12 1	0.09	9.5	0.10^S^	0.02 - 0.23
**R inferior temporal gyrus (p = 0.007)**	48 -17 -32	0.07	7.4	0.08^S^	0.004 - 0.21

f^2 S^ = small; f^2 M^ = moderate; f^2 L^ = large

Next, we evaluated whether the two cohorts differed in the level of sensorimotor and associative segregation. The S_longAG_ showed less segregated networks as compared with the S_shortAG_, but only for the Gordon parcellation scheme (F_1,194_ = 4.8, p = 0.03, δ = 0.69^M^, CI_95%_ [0.09 0.99]). When the same analyses were performed for each network, the LME model yielded a significant group × network interaction for both Gordon’s (F_11,1193_ = 3.6, p = 0.00004, f^2^ = 0.035^S^, CI_95%_[0.017 0.037]) and Ji’s parcellation scheme (F_11,1193_ = 3.4, p = 0.0001, f^2^ = 0.033^S^, CI_95%_[0.017 0.035]).

*Post hoc* analyses showed greater segregation of the cingulo-opercular network (CON) in the S_shortAG_ as compared to the S_longAG_ in both parcellation schemes (Gordon: F_1,93_ = 14.3, p = 0.003, f^2^ = 0.15^M^, CI_95%_[0.02 0.60]; Ji: F_1,93_ = 12.0, p = 0.009, f^2^ = 0.13^S^, CI_95%_[0.02 0.48]), as well as greater segregation of the mouth somatomotor network (mSMN) in Gordon’s parcellation (F_1,93_ = 12.7, p = 0.003, f^2^ = 0.14^S^, CI_95%_[0.03 0.35]) and greater segregation of the frontoparietal network (FPN) in Ji’s parcellation (F_1,93_ = 15.0, p = 0.002, f^2^ = 0.16^M^, CI_95%_[0.04 0.44]) when performing the same comparison. Figure 1 illustrates group differences for each functional network for Gordon’s parcellation (Fig. 1A) and Ji’s parcellation (Fig. 1B).

**Table 3:** LME models assessing the independent effect of *APOE* and HOMA-IR on drop error, and their interaction with subtask.

Model	Formulation of the LME model Dependent variable ~ predictor + covariates + (random effect for the intercept)
**1**	dropE ~ common covariates + (participants)
**2**	dropE ~ Subtask + common covariates + (participants)
**3**	dropE ~ HOMA-IR + Subtask + common covariates + (participants)
**4**	dropE ~ *APOE* + HOMA-IR + Subtask + common covariates + (participants)
**5**	dropE ~ HOMA-IR × *APOE* + Subtask + common covariates + (participants)
**6**	dropE ~ HOMA-IR × Subtask + HOMA-IR × *APOE* + common covariates + (participants)
**7**	dropE ~ *APOE* × Subtask + HOMA-IR × Subtask + HOMA-IR × *APOE* + common covariates + (participants)
**8**	dropE ~ HOMA-IR × *APOE* × Subtask + common covariates + (participants)

Note: dropE = drop error; ~ this symbol indicates the relationship between the dependent and independent variables. All models included age, education years, sex, BMI, MtbS score and incoming distance as common covariates.

**Figure 2.**
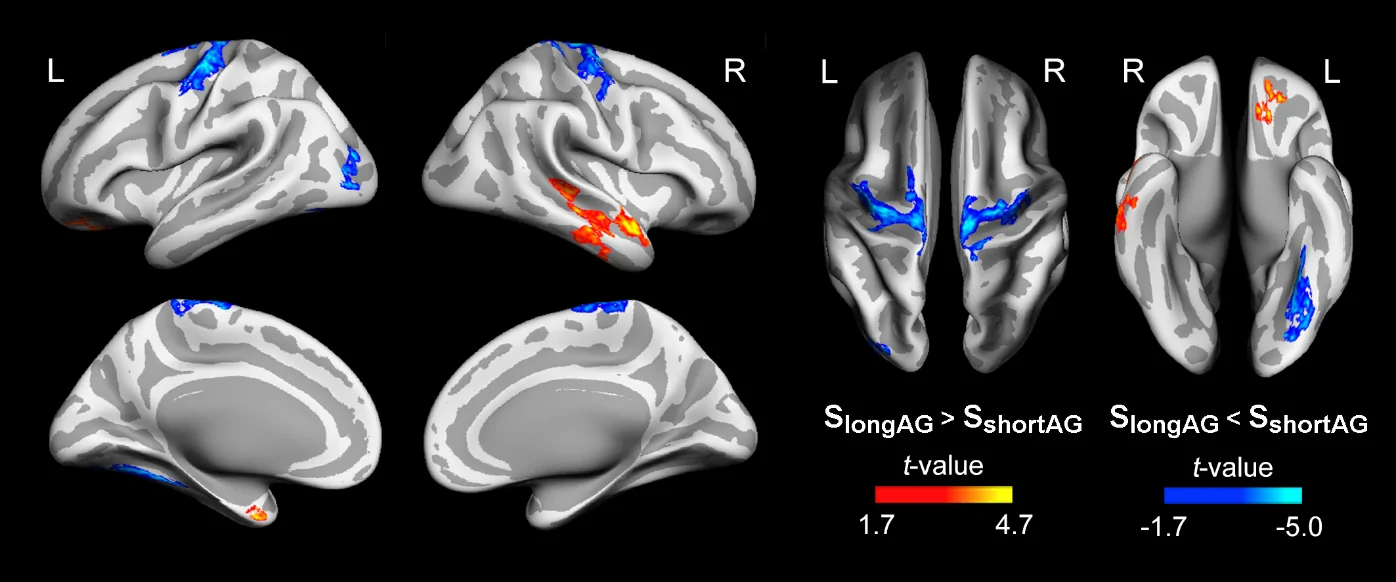
**Differences in cortical thickness between the two cohorts**. Vertex-wise significant differences in cortical thickness between the two cohorts after adjusting by age, gender, education years, BMI, and *APOE* genotype. Statistical results are summarized in Table 2.

**Figure 3.**
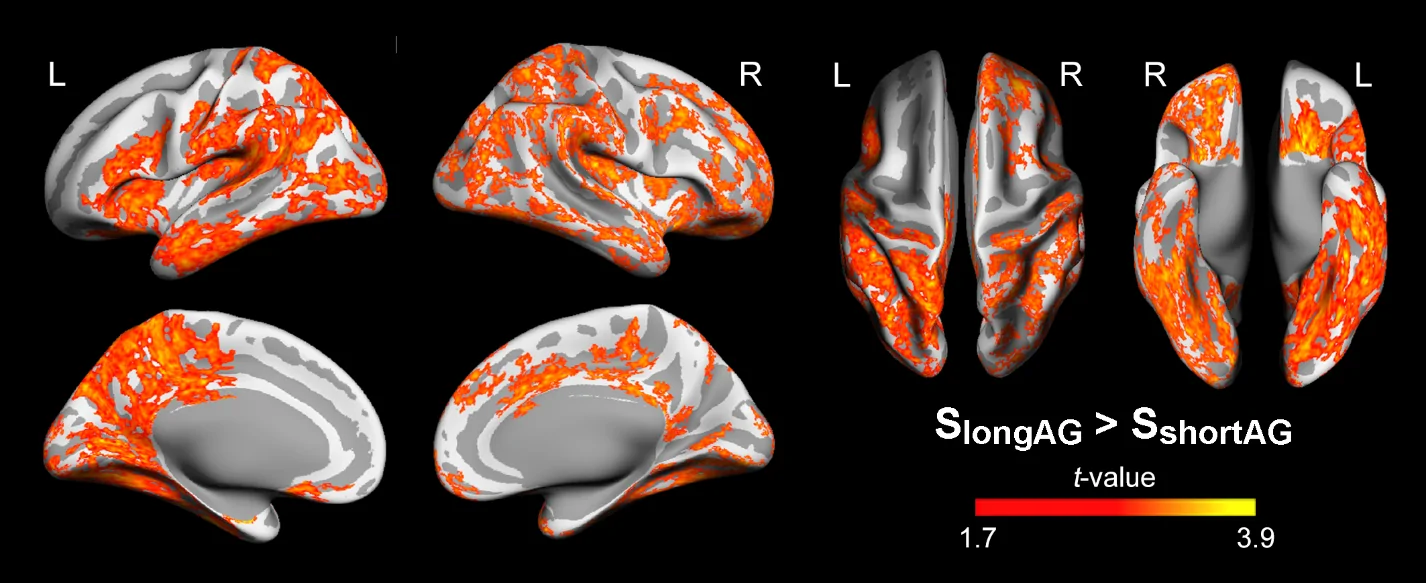
**Differences in intracortical myelin content between the two cohorts**. Vertex-wise significant differences in intracortical myelin content derived from the T1w/T2w ratio between the two cohorts after adjusting by age, gender, education years, BMI, and *APOE* genotype. Statistical results are summarized in Table 2.

Finally, we found that the two cohorts also differed in GM integrity (Fig. 2-3). A summary of results is included in Table 2. In comparison to the S_shortAG_, the S_longAG_ showed increased cortical thickness in the insula and superior temporal gyrus bilaterally, and in the superior frontal gyrus and pars opercularis of the right hemisphere, as well as cortical thinning in the left and right precentral gyrus (Fig. 2). Differences in T1w/T2w maps were highly distributed but always in the same direction (Fig. 3). The S_longAG_ showed higher values than the S_shortAG_ (Table 2).

In summary, compared to S_shortAG_, the S_longAG_ showed worse metabolic profile (Table 1), less segregated networks, particularly concerning the SMN, CON and FPN (Fig. 1), thickening in temporal and frontal areas and thinning in the motor cortex and visual regions (Fig. 2 and Table 2), as well as higher intracortical myelin content in regions highly distributed throughout the cortical mantle (Fig. 3 and Table 2).

### Influence of *APOE* and HOMA-IR on PI performance

We built eight individual LME models to determine in each sample the influence of subtask, *APOE* and HOMA-IR on drop error. The formulation of these models is shown in Table 3. Each model was compared with the immediately inferior model through an ANOVA. The significance of each predictor was assessed using the likelihood ratio test.

The model including the subtask as independent variable (i.e., model 2 in Table 3) provided a better fit to the data than the model only including the covariates (i.e., model 1 in Table 3) in the S_longAG_ (χ^2^ (2) = 126.4; p < 10^-15^, f^2^ = 0.024^S^, CI_95%_ [0.014 0.038]) and S_shortAG_ (χ^2^ (1) = 56.8; p < 10^-13^, f^2^ = 0.052^S^, CI_95%_ [0.028 0.092]). The drop error was lower in the LPI than in the PPI condition for both the S_longAG_ (p < 10^-7^) and S_shortAG_ (p < 10^-13^). In the S_longAG_, the drop error was also lower in the BPI than in the PPI condition (p < 10^-7^). No significant differences were found between the LPI and BPI conditions. The mean and SD of drop error for each virtual environment and cohort are shown in Table 4.

**Figure 4.**
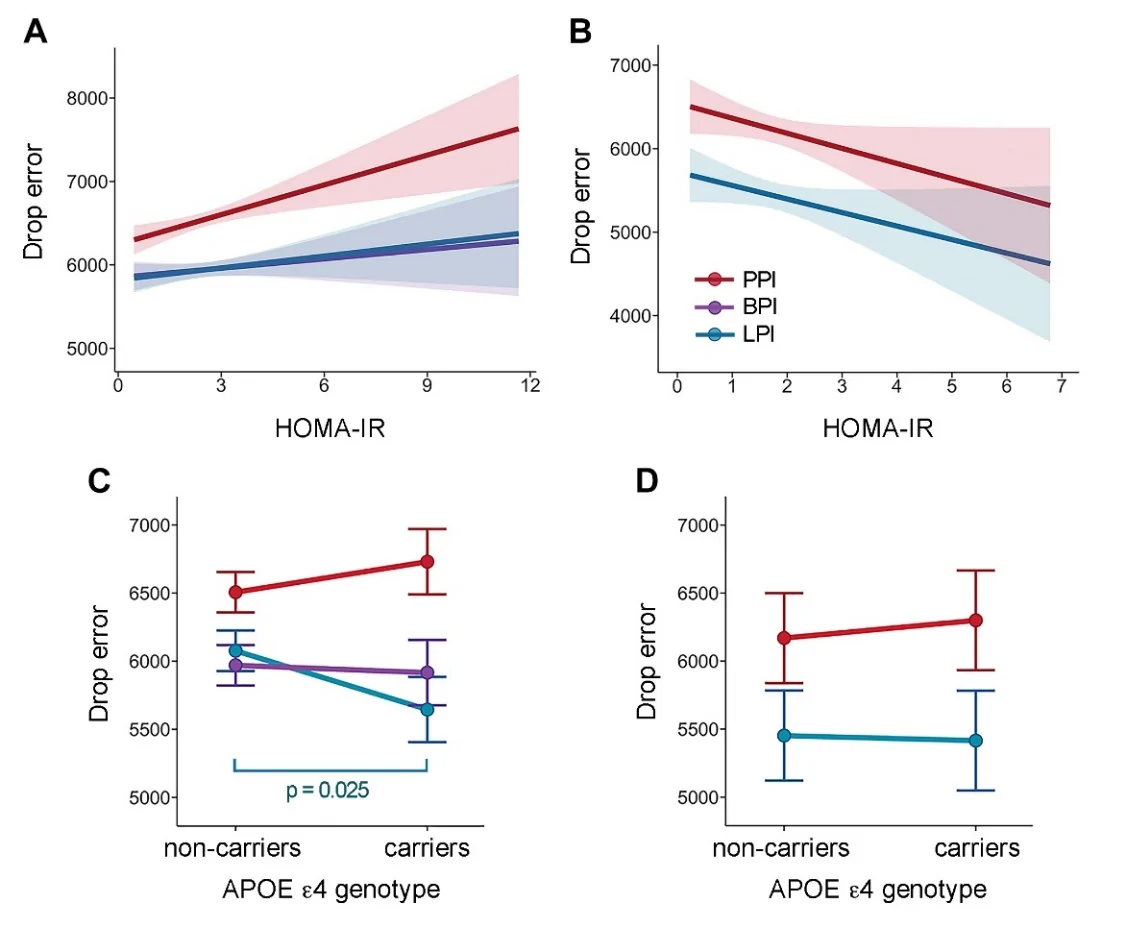
**Influence of *APOE* genotype and HOMA-IR on PI Performance across different subtasks**. (**A**) Scatterplots depicting the relationship between HOMA-IR in the S_longAG_ (N = 53) and drop errors across different subtasks. Significant associations were observed when combining subtasks, but not individually. (**B**) Scatterplots showing the relationship between HOMA-IR in the S_shortAG_ (N = 49) and drop errors across different subtasks. No significant associations were found either when subtasks were combined or individually. The shaded area around the regression slopes in (A) and (B) represents the 95% confidence interval. (**C**) Performance of S_longAG_ participants (which is inversely related to drop error) was notably enhanced in *APOE* ɛ4 carriers when supportive spatial cues were available, i.e. in the LPI subtask. (**D**) Performance of S_shortAG_ participants showed no significant impact of *APOE* genotype in either the PPI or LPI subtask.

Neither HOMA-IR (model 3 vs. model 2 in Table 3) nor *APOE* (model 4 vs. model 3 in Table 3) showed a main effect on drop error, nor was the interaction between these factors significant (model 5 vs. model 4 in Table 3). However, their effects were conditioned by the subtask. The HOMA-IR × subtask interaction term (model 6 vs. model 5 in Table 3) was significant only in the S_longAG_ (χ^2^ (2) = 7.02, p = 0.03, f^2^ = 0.029^S^, CI_90%_ [0.016 0.037]). The higher the HOMA-IR, the greater the drop error in the PPI condition in the S_longAG_ (F_1,53_ = 3.9, p = 0.05) but not in the S_shortAG_ (F_1,49_ = 2.5, p = 0.12). However, the correlation between the residuals of HOMA-IR and drop error in the S_longAG_ participants, after controlling for the effect of covariates, was not significant in the PPI condition (r = 0.02, p = 0.36). Therefore, we cannot conclude that the strength or direction of the association between HOMA-IR and drop error in the PPI subtask differed from that in the other subtasks. The scatterplots for these associations for both S_longAG_ and S_shortAG_ are shown in Figure 4A and 4B, respectively.

**Table 4:** Drop error for each subtask of the Apple Game in the two cohorts.

Subtask	S_longAG_ (N = 53) 32 trials per subtask 0-4 distractor trees	S_shortAG_ (N=49) 12 trials per subtask 1 distractor tree
**PPI**	4099.9 ± 2169.7	4336.2 ± 2044.6
**LPI**	3444.5 ± 2041.8	3556.1 ± 2028.7
**BPI**	3395.5 ± 1777.0	—

The *APOE* × subtask interaction (model 7 vs. model 6 in Table 3) was also significant in the S_longAG_ (χ^2^ (2) = 23.4, p = 0.0001, f^2^ = 0.029^S^, CI_90%_ [0.016 0.037]) but not in the S_shortAG_. As in our previous study [6], *APOE* ɛ4 carriers performed worse than non-carriers in the PPI subtask, but these differences did not reach statistical significance in the *post hoc* analyses (Fig. 4C). Interestingly, however, *APOE* ɛ4 carriers showed decreased drop error in the LPI condition when compared with non-carriers (p = 0.025; Fig. 4C)—again in line with our previous findings [6], although effects did not reach significance in that prior study. These results could not be replicated in the S_shortAG_ (Fig. 4D). One possibility for the inconsistency across samples is that the S_shortAG_ performed the task with only one level of difficulty. To test this hypothesis, we repeated the analyses in the S_longAG_ while retaining only those trials that included a single distractor tree. The interaction term *APOE* × subtask was not significant after removing variability in the number of distractor trees, indicating that a wider range of difficulty is required to unmask the effect of *APOE* genotype when spatial cues are unavailable. The effect of *APOE* in the LPI condition was also not significant with only one level of difficulty.

We also assessed whether the effect of *APOE* on drop error across subtasks was moderated by HOMA-IR (model 8 vs. model 7 in Table 3), but the three-way interaction was not significant.

### Association between network segregation and PI performance

To investigate the effects of network segregation on drop error, either in isolation or in interaction with other predictor variables, different LME models were designed, the formulation of which is shown in Table 5. To assess the relationship between the segregation index and PI estimates, we applied model 1 included in Table 5. No significant association was found for either the sensorimotor or the associative network in either sample. Only the S_shortAG_ showed a significant association between drop error and functional segregation for visual attention network 2 (VN2) (β = -0.15, F_1,1165_ = 28.4, p < 10^-5^, f^2^ = -0.11^S^, CI_95%_[-0.12 -0.002]) and FPN in the Ji parcellation scheme (β = -0.12, F_1,1165_ = 18.0, p = 0.0003, f^2^ = -0.118^S^, CI_95%_[-0.121 -0.119]). In both cases, the larger the segregation index, the lower the drop error, and therefore, the better the estimation of PI. This association was not moderated by the presence/absence of spatial cues in either of the cohorts (i.e., model 2 in Table 5 yielded no significant results).

**Table 5:** LME models applied to assess the moderating effect of brain network segregation on the influence of HOMA-IR and *APOE* on drop error.

Model	Formulation of the LME model Dependent variable ~ predictor + covariates + (random effect for the intercept)
**1**	dropE ~ seg + HOMA-IR + *APOE* + Subtask + common covariates + (participants)
**2**	dropE ~ seg × Subtask + HOMA-IR + *APOE* + common covariates + (participants)
**3**	dropE ~ seg × HOMA-IR + *APOE* + Subtask + common covariates + (participants)
**4**	dropE ~ seg × HOMA-IR × Subtask + *APOE* + common covariates + (participants)
**5**	dropE ~ seg × *APOE* + HOMA-IR + Subtask + common covariates + (participants)
**6**	dropE ~ seg × *APOE* × Subtask + HOMA-IR + common covariates + (participants)

Note: dropE = drop error; ~ this symbol indicates the relationship between the dependent and independent variables; seg = segregation index for each network in the Gordon or Ji parcellation scheme. All models included age, education years, sex, BMI, MtbS score and incoming distance as common covariates. In those cases where segregation was computed for averaged sensorimotor and associative networks, the type of network was also included as covariate.

### Effect of HOMA-IR and *APOE* on network segregation and FC within and between networks

We first assessed whether HOMA-IR and *APOE* explained some of the observed variability in the level of segregation of sensorimotor and associative networks. The LME model yielded no significant main effect of HOMA-IR on the level of system segregation. However, HOMA-IR did influence FC (average of FC within and between networks) in Gordon’s parcellation (β = 0.11, F_1,398_ = 5.2, p = 0.02, f^2^ = -0.002, CI_95%_[-0.007 -10^-7^]) and Ji’s parcellation (β = 0.14, F_1,398_ = 8.6, p = 0.004, f^2^ = -0.009, CI_95%_[-0.03 -0.004]), but both size effects were negligible (f^2^ < 0.02).

The next step was to assess whether the strength of the association between HOMA-IR and system segregation was influenced by whether FC was measured within-network or between-networks. The interaction was significant for Gordon’s parcellation (β = 0.08, F_1,397_ = 7.2, p = 0.008, ρ = -0.53^S^, CI_95%_[-1.00 0.12]). The HOMA-IR was significantly correlated with between-FC (β = 0.28, F_1,195_ = 7.9, p = 0.005, f^2^ = -0.01, CI_95%_[-0.03 -0.01]) but not with within-FC. Note, however, that the size effect for the former was negligible (f^2^ < 0.02). For Ji’s parcellation, the interaction was also significant (β = 0.10, F_1,397_ = 9.3, p = 0.002, ρ = -0.60^S^, CI_95%_[-1.10 -0.03]). In this particular case, the HOMA-IR was significantly associated with both within-FC (β = 0.15, F_1,195_ = 4.0, p = 0.048, f^2^ = -0.01, CI_95%_[-0.04 -0.004]) and between-FC (β = 0.41, F_1,195_ = 16.9, p = 0.00006, f^2^ = -0.02^S^, CI_95%_[-0.004 -0.002]), but the size effect for the former was negligible. These findings imply that insulin resistance, as measured by HOMA-IR, might contribute to changes in brain connectivity, particularly affecting how different brain networks interact.

We found no significant differences between carriers and non-carriers of *APOE* ɛ4 regarding network segregation levels. However, *APOE* had an overall effect on both within- and between-FC. *APOE* ɛ4 carriers exhibited lower FC than non-carriers (F_1,398_ = 5.2, p = 0.02, δ = -0.70^M^, CI_95%_ [-1.37 0.22]). Note that while the standardized local effect size was moderate, the CI_95%_ included zero, which requires some caution in its interpretation. This effect was independent of whether FC was evaluated within or between networks, which might explain why there were no differences in segregation level between *APOE* ɛ4 carriers and non-carriers.

### Network segregation moderates the impact of HOMA-IR on PI performance

**Figure 5.**
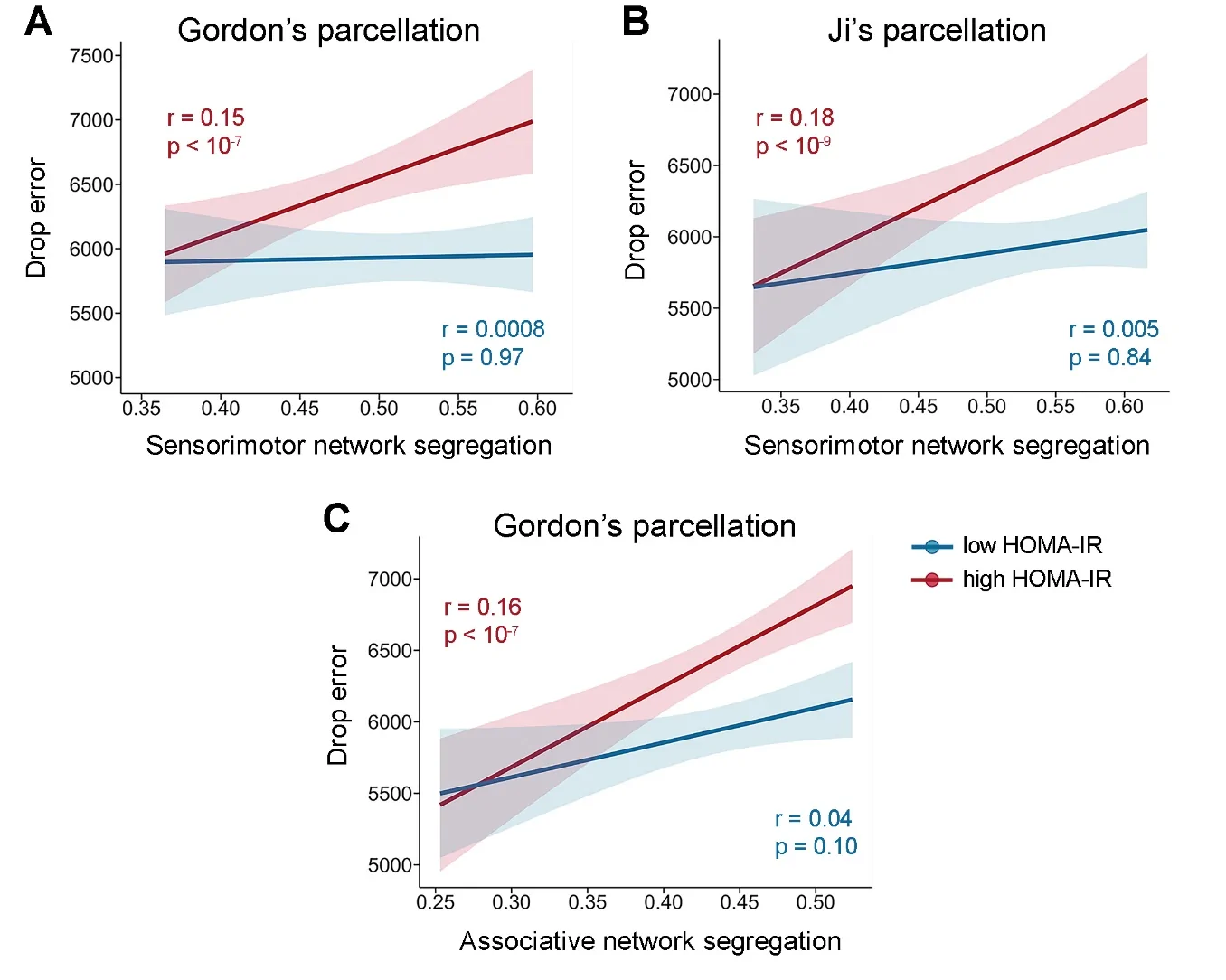
**Association between network segregation and PI as influenced by HOMA-IR**. (**A**) Scatterplots depicting the relationship between sensorimotor network segregation in the Gordon parcellation scheme and drop error among individuals of the S_longAG_ (N = 53) with varying HOMA-IR levels. (**B**) Similar analysis as in (A) but for the sensorimotor network in the Ji parcellation scheme. (**C**) Similar analysis as in (A) but for the associative network in the Gordon parcellation scheme. The shaded area around the regression slopes represents the 95% confidence interval. Note that in all cases, the relationship was significant for individuals with higher HOMA-IR values.

The moderating role of network segregation on the relationship between HOMA-IR and PI (model 3 in Table 5) was evident only in the S_longAG_, which exhibited higher values for this variable. The effect was significant for both the sensorimotor network in Gordon’s (β = 0.08, F_1,5075_ = 38.1, p < 10^-9^, ρ = -0.15^S^, CI_95%_[0.10 -0.19]) and Ji’s parcellation (β = 0.07, F_1,5075_ = 49.5, p < 10^-11^, ρ = 0.14^S^, CI_95%_[0.10 0.18]), and the associative network in the Gordon scheme (β = 0.09, F_1,5075_ = 44.5, p < 10^-10^, ρ = 0.17^S^, CI_95%_[0.12 0.22]). The results of *post hoc* analyses are illustrated in Figure 5. Note that the loss of segregation of both the sensorimotor network and the associative network was beneficial for PI processes in individuals with higher insulin resistance.

**Table 6:** Moderating role of functional network segregation on the relationship between HOMA-IR and drop error.

S_longAG_ (N=53)						S_shortAG_ (N=49)					

**Network**	*β*	*F_1,5075_*	*p*	ρ	*CI_95%_*	Network	*β*	*F_1,1164_*	*p*	*ρ*	*CI_95%_*
***Gordon’s parcellation***									
**VN**	0.07	49.1	10^-10^	0.14^S^	0.10 - 0.18	mSMN	0.12	9.9	0.01	0.22^S^	0.11 - 0.36
**FPN**	0.09	35.3	10^-8^	0.17^S^	0.12 - 0.23	DMN	0.20	16.8	0.0005	0.37^S^	0.24 - 0.56
**SN**	0.09	40.9	10^-9^	0.16^S^	0.11 - 0.21	CON	0.10	9.1	0.01	0.18^S^	0.09 - 0.31
**CPN**	0.11	23.7	10^-5^	0.21^S^	0.13 - 0.30						
***Ji’s parcellation***									
**VN1**	0.09	38.5	10^-9^	0.17^S^	0.12 - 0.23	DAN	0.14	14.5	0.001	0.26^S^	0.17 - 0.39
**SMN**	0.08	53.2	10^-11^	0.15^S^	0.11 - 0.19	VMN	0.15	16.1	0.0007	0.27^S^	0.17 - 0.41
**PMN**	0.10	29.0	10^-6^	0.19^S^	0.12 - 0.27	PMN	-0.11	20.4	0.0001	-0.20^S^	-0.29 - -0.13
						CON	0.12	16.1	0.008	0.23^S^	0.12 - 0.37

ρ^S^ = small; ρ^M^ = moderate; ρ^L^ = large; shaded rows = similarities between the two samples

Analyses performed in specific networks yielded significant results in the two cohorts (Table 6). As might be expected from sample differences in the metabolic profile, the specific sensorimotor and associative networks affected in each case were different with one exception, the posterior multimodal network (PMN) in the Ji parcellation scheme. Nevertheless, we found differences in *post hoc* analyses. While participants of the S_longAG_ with the highest HOMA-IR values showed a positive association between PMN segregation and drop error, the opposite was true in the other sample. The contradiction is only apparent because if we look at the distribution of HOMA-IR values in the two cohorts, we can see that the highest values in the S_longAG_ range between 3.2 and 11.7, while in the S_shortAG_, they range between 2.2 and 6.8. Taken together, the results indicate that individuals with intermediate HOMA-IR values (i.e., high HOMA-IR in the S_shortAG_) might benefit from the PMN segregation in estimating PI, while those with higher values (i.e., high HOMA-IR in the S_longAG_) might benefit more from the integration of this network with the rest of the brain.

Next, we investigated whether the level of sensorimotor and/or associative segregation influenced the effect of HOMA-IR on PI across different subtasks (model 4 in Table 5). The three-way interaction was not significant for either the sensorimotor or associative networks. The same analysis was conducted for every single network and obtained the same results.

**Figure 6.**
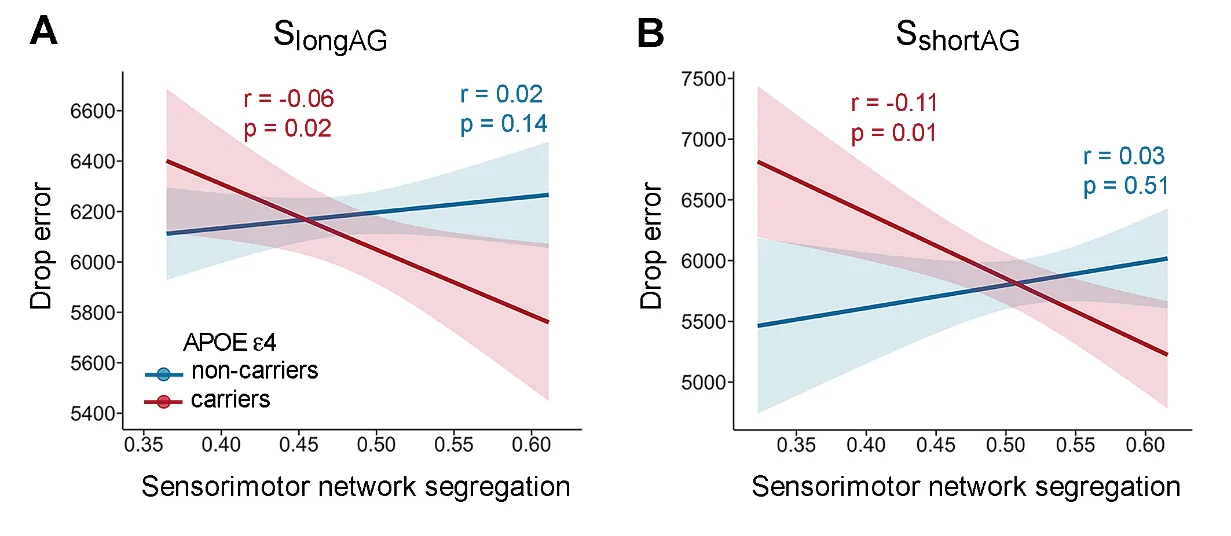
**Association between sensorimotor network segregation and PI across the two cohorts**. (**A**) Scatterplots illustrating the relationship between sensorimotor network segregation in the Gordon parcellation scheme and drop error in S_longAG_ for *APOE* ε4 carriers (n = 15) and non-carriers (n = 38). (**B**) Similar analysis as in (A) but for S_shortAG_ (ε4 carriers = 22; non-carriers = 27). The shaded area around the regression slope represents the 95% confidence interval. Note that the relationship was significant only for ε4 carriers.

**Table 7:** Significant results derived from model 3 and 5 in Table 5 for each network, parcellation scheme and cohort.

Segregation type (cohort) Factor × Network seg	Scheme	β	*F_DF_*	*p*	Size effect ρ	*CI_95%_*
***Seg_Thickness_ (S_longAG_, N = 53)***			*F_1,5075_*			
**HOMA-IR × SMN seg**	Ji	0.07	48.7	10^-11^	0.14^S^	0.10 - 0.18
**HOMA-IR × ASN seg**	Gordon	0.08	45.1	10^-10^	0.16^S^	0.11 - 0.21
	Ji	0.07	43.1	10^-10^	0.14^S^	0.10 - 0.18
***Seg_Myelin_ (S_longAG_, N = 53)***			*F_1,5075_*			
**HOMA-IR × SMN seg**	Gordon	-0.10	17.4	0.00006	-0.24^S^	-0.29 - -0.11
**HOMA-IR × ASN seg**	Gordon	-0.20	14.4	0.0001	-0.39^S^	-0.60 - -0.19
	Ji	-0.21	46.4	10^-10^	-0.40^S^	-0.52 - -0.28

Seg_Thickness_ = network segregation adjusted by cortical thickness; Seg_Myelin_ = network segregation adjusted by intracortical myelin; ρ^S^ = small; ρ^M^ = moderate; ρ^L^ = large; SMN = sensorimotor network; ASN = associative network. DF=Degrees of freedom.

### Network segregation moderates the impact of *APOE* on PI performance across subtasks

The level of network segregation did moderate the association between *APOE* and drop error (model 5 in Table 5). Specifically, it was the sensorimotor network, not the associative one, that acted as a moderator in both the S_longAG_ (Gordon: β = 0.10, F_1,5075_ = 9.7, p = 0.004, δ = 0.20^S^, CI_95%_[0.07 0.32]) and S_shortAG_ (Gordon: β = 0.22, F_1,1164_ = 13.4, p = 0.0005, δ = 0.47^S^, CI_95%_[0.19 0.71]; Ji: β = 0.21, F_1,1164_ = 13.5, p = 0.0005, δ = 0.45^S^, CI_95%_[0.16 0.67]). Results of *post hoc* analyses shown in Figure 6 indicated that carriers of the ɛ4 allele benefited most from the segregation of the sensorimotor network when estimating PI.

Upon analyzing individual networks, it was observed that PI in the ɛ4 carriers of the S_longAG_ benefited from the segregation of the VN2 according to Ji's parcellation (β = -0.19, F_1,5075_ = 46.8, p < 10^-9^, δ = 0.40^S^, CI_95%_[0.28 0.51]). While in the S_shortAG_ ɛ4-carrier participants, PI was associated with less segregated associative networks such as the ventral attention network (VAN) (β = 0.27, F_1,1164_ = 21.6, p = 0.0004, δ = 0.60^S^, CI_95%_[0.29 0.84]) and CON (β = 0.20, F_1,1164_ = 9.5, p = 0.008, δ = 0.43^S^, CI_95%_[0.12 0.67]) in Gordon’s parcellation and the FPN (β = 0.20, F_1,1164_ = 11.7, p = 0.008, δ = 0.43^S^, CI_95%_[0.12 0.67]) in Ji’s parcellation.

Further analyses were conducted to examine whether the level of sensorimotor and/or associative segregation influenced the impact of *APOE* on PI as a function of availability of spatial cues in the different virtual environments (model 6 in Table 5). Each type of segregation was assessed separately because the sample size does not allow us to evaluate a four-way interaction. A significant three-way interaction was found for sensorimotor but not for associative segregation in S_longAG_ participants, both in Gordon's parcellation (F_2,5035_ = 3.6, p = 0.026, f^2^ = 0.03^S^, CI_95%_[0.005 0.07]) and Ji's parcellation (F_2,5035_ = 3.6, p = 0.026, f^2^ = 0.03^S^, CI_95%_[0.005 0.07]). Subsequently, we stratified the sample based on tertiles of sensorimotor segregation and focused on extreme values for *post hoc* analyses. Among individuals exhibiting the highest levels of sensorimotor segregation, *APOE* ɛ4 carriers demonstrated superior performance (5325.9 ± 1846.7) compared to non-carriers (5936.0 ± 1854.9), specifically in the LPI condition (p = 0.0006). We applied the same approach to VN2 using Ji's parcellation. The three-way interaction did not reach significance. Nonetheless, we conducted *post hoc* analyses to explore whether the level of segregation of this network contributed to explain why, in this sample, ɛ4 carriers did not exhibit worse performance than non-carriers in the absence of spatial cues, as previously demonstrated [6]. We only found a significant *APOE* ×subtask interaction among individuals exhibiting the lowest segregation levels in this network (F_2,1710_ = 11.5, p = 0.00001). Among these individuals, ɛ4 carriers performed worse (7188.3 ± 1797.2) than non-carriers (6353.3 ± 1955.9) in the PPI condition (p = 0.0007), while no significant differences were found for the remaining subtasks.

### GM integrity influences the moderating role of network segregation on the association of HOMA-IR and *APOE* with PI performance

As GM integrity differed between the two cohorts, we next assessed whether the moderating role of network segregation on the relationship of HOMA-IR and *APOE* with PI still holds after removing the contribution of cortical thickness and intracortical myelin from the computed FC between the nodes of the different networks. For this purpose, we applied models 3 and 5 included in Table 5 on the computed individual-level segregation after removing the variability in cortical thickness and T1w/T2w ratio. The two models yielded significant results, but only for HOMA-IR (see Table 7).

**Table 8:** Moderating effects of network segregation on the association of *APOE* and HOMA-IR with PI adjusted by the influence of GM integrity.

	S_longAG_ (N = 53)			S_shortAG_ (N = 49)		

**Factor**	Seg_Raw_	Seg_Thickness_	Seg_Myelin_	Seg_Raw_	Seg_Thickness_	Seg_Myelin_
***APOE***	SMN	—	—	SMN	—	—
**HOMA-IR**	SMN, ASN	SMN, ASN	SMN, ASN	—	—	—

SMN = sensorimotor network; ASN = associative network; Seg_Raw_ = original network segregation; Seg_Thickness_ = network segregation adjusted by cortical thickness; Seg_Myelin_ = network segregation adjusted by intracortical myelin.

**Figure 7.**
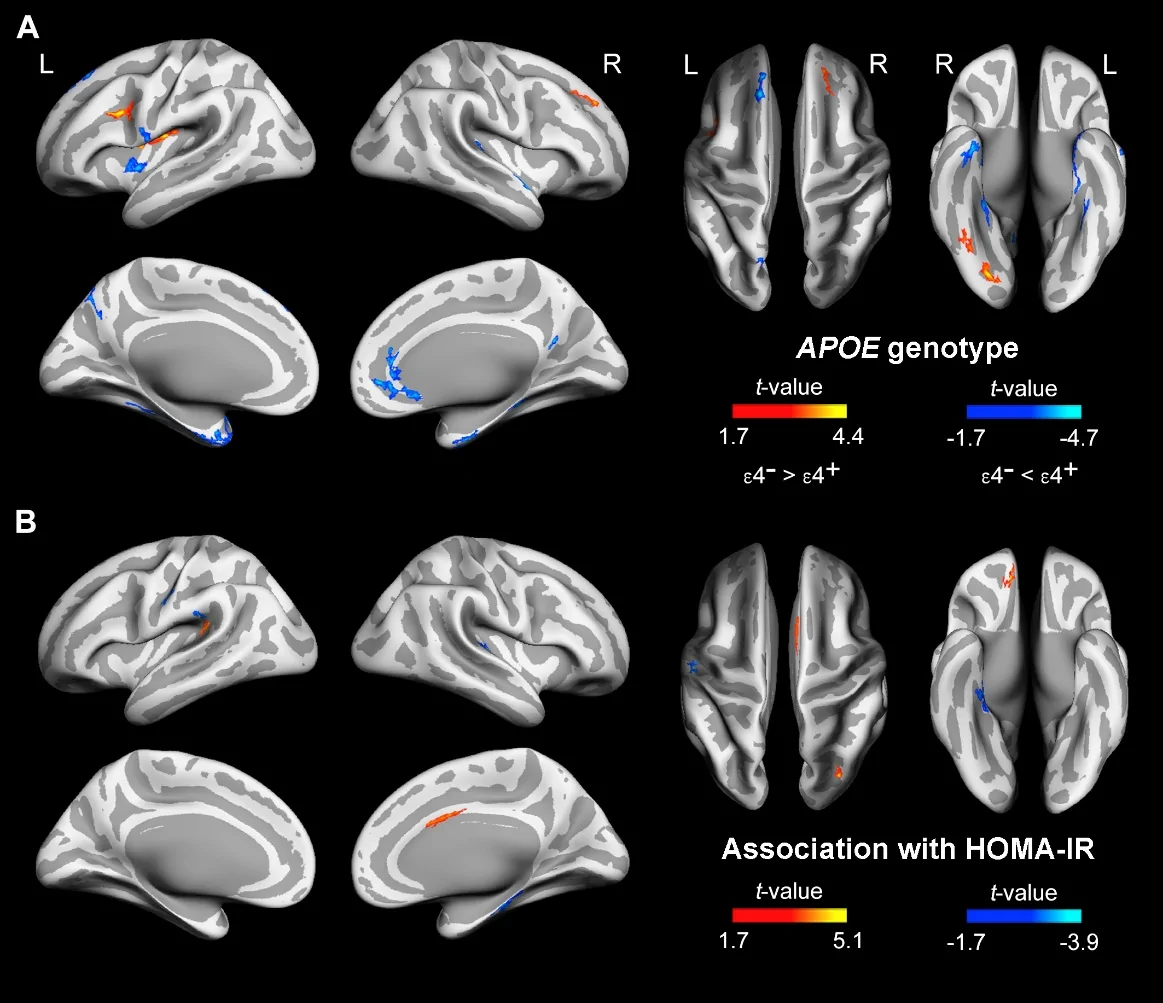
**Impact of *APOE* genotype and HOMA-IR on cortical thickness**. (**A**) Differences in cortical thickness between *APOE* ɛ4 carriers (ɛ4^+^) and non-carriers (ɛ4^-^). Regions in red indicate cortical thinning and regions in blue cortical thickening in *APOE* ɛ4 carriers compared to noncarriers. (**B**) Association between cortical thickness and HOMA-IR. Regions in red indicate a positive association, while regions in blue indicate a negative association. Statistical results are summarized in Table 9.

**Figure 8.**
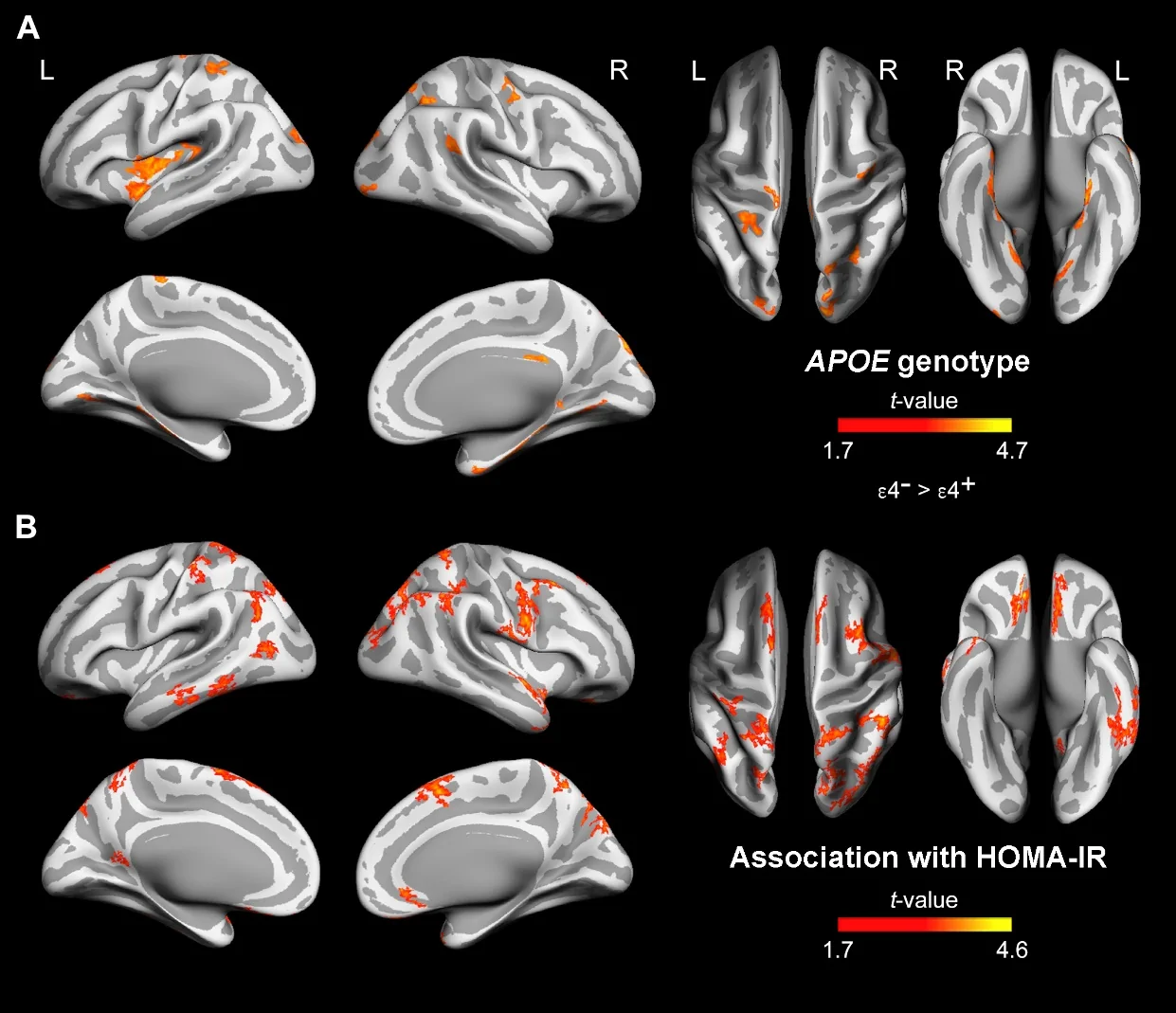
**Impact of HOMA-IR and *APOE* genotype on intracortical myelin**. (**A**) Differences in intracortical myelin, as derived from the T1w/T2w ratio, between *APOE* ɛ4 carriers (ɛ4^+^) and non-carriers (ɛ4^-^). Regions in red indicate decreased intracortical myelin in APOE ɛ4 carriers compared to noncarriers. (**B**) Association between intracortical myelin content and HOMA-IR. Regions in red indicate a positive association with HOMA-IR. Statistical results are summarized in Table 10.

Table 8 describes how cortical thickness and intracortical myelin influence the moderating effect of functional network segregation on the impact of APOE and HOMA-IR on PI performance. Interestingly, the moderating effect of network segregation disappeared for the APOE genotype after removing the contribution of variability in GM integrity from the FC (i.e., both cortical thickness and intracortical myelin). This result suggests that the beneficial effect of sensorimotor segregation on PI in APOE ɛ4 carriers may be driven by differences in cortical thickness and intracortical myelin content. In line with this hypothesis, we found that in comparison with APOE ɛ4 non-carriers, APOE ɛ4 carriers showed cortical thickening in specific regions of lateral frontal and cingulate cortices and cortical thinning in medial frontal, medial temporal and medial parietal regions (Fig. 7A and Table 9). Similarly, APOE ɛ4 carriers showed highly distributed decreases in the T1w/T2w ratio when compared with non-carriers (Fig. 8A and Table 9).

**Table 9:** Impact of the *APOE* genotype on the GM integrity.

*GM measurement* Contrast Brain cortical region (p_cluster_)	MNI	R^2^	F_9,95_	Size effect f^2^	CI_95%_
***Cortical thickness*** **ɛ4^-^ > ɛ4^+^**					
**L insula (p = 0.001)**	-35 -13 18	0.12	14.2	0.15^S^	0.03 - 0.37
**L caudal middle frontal gyrus (p = 0.04)**	-33 12 28	0.13	14.5	0.15^M^	0.01 - 0.41
**R fusiform gyrus (p = 0.00001)**	28 -76 -9	0.18	22.5	0.24^M^	0.07 - 0.49
**R superior frontal gyrus (p = 0.00001)**	18 44 36	0.12	14.2	0.15^S^	0.03 - 0.36
**ɛ4^-^ < ɛ4^+^**					
**L temporal pole (p = 0.00004)**	-22 7 -36	0.10	10.7	0.11^S^	0.01 - 0.31
**L precuneus (p = 0.008)**	-4 -73 45	0.10	10.6	0.11^S^	0.02 - 0.29
**L insula (p = 0.009)**	-40 -4 0	0.11	12.6	0.13^S^	0.02 - 0.34
**L superior frontal gyrus (p = 0.01)**	-11 33 52	0.16	19.0	0.20^M^	0.04 - 0.46
**L superior temporal gyrus (p = 0.02)**	-46 17 -20	0.16	19.5	0.20^M^	0.06 - 0.44
**L fusiform gyrus (p = 0.02)**	-33 -32 -26	0.10	10.9	0.11^S^	0.02 - 0.30
**L precentral gyrus (p = 0.03)**	-59 -2 12	0.12	13.8	0.14^S^	0.03 - 0.32
**R medial orbitofrontal gyrus (p = 0.0003)**	12 42 -3	0.11	12.2	0.13^S^	0.03 - 0.30
**R fusiform gyrus (p = 0.001)**	30 0 -43	0.13	15.1	0.16^M^	0.03 - 0.37
**R rostral anterior cingulate gyrus (p = 0.002)**	7 43 11	0.10	10.6	0.11^S^	0.01 - 0.28
**R superior temporal gyrus (p = 0.005)**	57 7 -10	0.11	11.9	0.13^S^	0.02 - 0.31
**R entorhinal cortex (p = 0.007)**	21 -2 -34	0.12	13.3	0.14^S^	0.04 - 0.31
**R parahippocampal gyrus (p = 0.03)**	28 -32 -16	0.10	11.0	0.12^S^	0.02 - 0.30
**R precuneus (p = 0.04)**	3 -57 21	0.14	15.8	0.17^M^	0.04 - 0.38
**R transverse temporal gyrus (p = 0.05)**	42 -23 13	0.10	10.7	0.11^S^	0.01 - 0.32
***Intracortical myelin (T1w/T2w)***					
**ɛ4^-^ > ɛ4^+^**					
**L parahippocampal cortex (p = 0.0000002)**	-18 -23 -20	0.18	22.6	0.24^M^	0.10 - 0.44
**L superior parietal cortex (p = 0.002)**	-8 -93 24	0.12	13.6	0.14^S^	0.04 - 0.30
**L isthmus cingulate cortex (p = 0.009)**	-6 -41 23	0.13	15.2	0.16^M^	0.05 - 0.34
**L insula (p = 0.01)**	-38 -9 -6	0.13	15.2	0.16^M^	0.05 - 0.33
**L lingual gyrus (p = 0.04)**	-6 -73 -4	0.12	13.8	0.14^S^	0.04 - 0.31
**R parahippocampal cortex (p = 0.0000002)**	19 -26 -19	0.17	20.2	0.21^M^	0.07 - 0.42
**R superior parietal cortex (p = 0.0000003)**	10 -84 39	0.15	18.0	0.19^M^	0.05 - 0.38
**R isthmus cingulate cortex (p = 0.00005)**	14 -38 -3	0.14	16.5	0.17^M^	0.05 - 0.35
**R entorhinal cortex (p = 0.00007)**	22 5 -32	0.17	20.1	0.21^M^	0.09 - 0.43
**R posterior cingulate cortex (p = 0.003)**	5 -27 26	0.14	15.8	0.17^M^	0.05 - 0.34
**R pericalcarine fisure (p = 0.007)**	9 -90 6	0.13	14.3	0.15^M^	0.05 - 0.32
**R lingual gyrus (p = 0.007)**	15 -60 -5	0.12	14.0	0.15^S^	0.04 - 0.32

f^2 S^ = small; f^2 M^ = moderate; f^2 L^ = large

The moderating effect of network segregation for HOMA-IR was maintained after removing the influence of GM integrity and was only evident in the S_longAG_. These results indicate that the moderating effect of network segregation on the association between insulin resistance and PI is independent of GM variability, even though HOMA-IR may contribute to such variability. Indeed, HOMA-IR was associated with increases and decreases in cortical thickness (Fig. 7B and Table 10) and with increases in T1w/T2w ratio (Fig. 8B and Table 10).

## DISCUSSION

This study aimed to investigate whether carriers of the *APOE* ε4 allele and individuals with elevated peripheral insulin resistance exhibit similar impairments in PI and how brain network specialization during the resting state modulates the impact of these AD risk factors on PI processes. Contrary to our initial hypothesis, we found that the effects of *APOE* ε4 and insulin resistance on PI differed, operating through distinct large-scale network mechanisms. Specifically, individuals with higher insulin resistance performed better in PI tasks when their brain networks were less segregated. This effect was observed regardless of spatial cue availability or gray matter integrity. In contrast, *APOE* ε4 carriers relied more heavily on cortical integrity and spatial landmarks for accurate navigation. Notably, ε4 carriers outperformed non-carriers when local landmarks were present, a performance linked to increased segregation within sensorimotor networks.

The study included two cohorts, S_shortAG_ and S_longAG_, and our results revealed significant differences between them. The S_longAG_ cohort exhibited a worse metabolic profile, greater GM atrophy, and less segregated brain networks compared to the S_shortAG_ cohort. This reduced network segregation in the S_longAG_ group may reflect compensatory cognitive strategies in response to metabolic impairments, potentially leading to cognitive overload and difficulty in maintaining coherent spatial representations. In contrast, the S_shortAG_ cohort showed enhanced network segregation, which seems to facilitate more focused cognitive processing and supports more accurate PI estimation.

Overall, these findings underscore the importance of understanding the complex interplay between metabolic health, genetic predisposition, and neural integrity. These factors collectively shape individual differences in cognitive performance and highlight the need for further research into how these mechanisms contribute to variability across populations.

**Table 10.** Association of HOMA-IR with the GM integrity.

*GM measurement* Contrast Brain cortical region (p_cluster_)	MNI	R^2^	F_10,94_	Size effect f^2^	CI_95%_
***Cortical thickness*** **Positive association with HOMA-IR**					
**L supramarginal gyrus (p = 0.04)**	-42 -40 22	0.09	10.2	0.11^S^	0.01 - 0.34
**R lateral orbitofrontal gyrus (p = 0.003)**	11 45 -22	0.15	17.6	0.19^M^	0.22 - 0.53
**R caudal anterior cingulate gyrus (p = 0.01)**	4 7 32	0.10	10.6	0.11^S^	0.01 - 0.33
**R inferior parietal gyrus (p = 0.04)**	36 -77 21	0.20	25.8	0.27^M^	0.06 - 0.75
**Negative association with HOMA-IR**					
**L postcentral gyrus (p = 0.009)**	-53 -13 41	0.10	10.6	0.11^S^	0.02 - 0.28
**L supramarginal gyrus (p = 0.04)**	-52 -32 20	0.11	12.6	0.13^S^	0.01 - 0.39
**R transverse temporal gyrus (p = 0.03)**	39 -22 10	0.14	15.8	0.17^M^	0.03 - 0.42
**R parahippocampal gyrus (p = 0.04)**	21 -22 -25	0.09	10.4	0.11^S^	0.02 - 0.31
***Intracortical myelin (T1w/T2w)***					
**Positive association with HOMA-IR**					
**L inferior temporal gyrus (p = 0.00002)**	-58 -48 -13	0.14	16.4	0.17^S^	0.01 - 0.98
**L medial orbitofrontal cortex (p = 0.0001)**	-7 18 -23	0.10	10.6	0.11^S^	0.01 - 0.42
**L superior parietal gyrus (p = 0.001)**	-29 -52 66	0.11	12.3	0.13^S^	0.01- 0.33
**L postcentral gyrus (p = 0.05)**	-29 -31 60	0.10	11.1	0.12^S^	0.01 - 0.25
**R supramarginal gyrus (p = 0.00005)**	49 -40 42	0.12	14.8	0.15^S^	0.01 - 0.44
**R lateral orbitofrontal gyrus (p = 0.006)**	14 29 -22	0.17	21.0	0.22^M^	0.03 - 0.56
**R precentral gyrus (p = 0.007)**	59 0 29	0.11	11.9	0.13^S^	0.01 - 0.35

f^2 S^ = small; f^2 M^ = moderate; f^2 L^ = large

### Distinct large-scale network mechanisms underlie the effects of *APOE* ɛ4 and insulin resistance on PI performance

Previous evidence indicates that older adults at higher risk for AD struggle more with accurate PI estimation when compensatory strategies are unavailable [2,6,61]. Overall, we did not observe an *APOE*-related effect on PI performance in the absence of supportive spatial cues. Nonetheless, ε4 carriers with less segregation in the secondary visual network (VN2 in Ji's parcellation) demonstrated poorer PPI estimates. Conversely, individuals with high insulin resistance exhibited a non-significant trend in this aspect. Unlike ε4 carriers, however, their PI estimation was negatively impacted by reduced segregation in both sensorimotor and associative networks, irrespective of spatial cue availability.

The results in ε4 carriers during the PPI condition were derived from secondary analyses and thus require cautious interpretation. They suggest that accurate information processing within VN2 may mitigate grid cell dysfunction in ε4 carriers, but not in insulin-resistant individuals. A pathway linking V2 to layer 5a of the entorhinal cortex [62], projecting to the CA1 region of the hippocampus [63], supports this interpretation. Notably, the level of VN2 segregation appears unaffected by *APOE* ε4 or insulin resistance, suggesting potential integrity of the visual cortical-entorhinal pathway in ε4 carriers but possible compromise in insulin-resistant individuals. Alternatively, the hippocampus might face challenges in integrating information from this pathway and other brain systems in insulin-resistant individuals compared to ε4 carriers. Supporting this view, recent evidence from microRNAs in extracellular vesicles derived from adipose tissue has revealed synaptic loss in the hippocampus and cognitive decline in high-fat diet-fed mice and diabetic patients [64]. Although synaptic density may also be reduced in the hippocampus of ε4 carriers, this effect is primarily linked to tau pathology and, to a lesser extent, to Aβ load [65], which may help explain discrepancies noted in earlier studies [6]. It is plausible that previous investigations included a higher proportion of ε4 carriers with AD-associated pathology compared to our study. Despite these discrepancies, our findings suggest that ε4 carriers and insulin-resistant individuals employ distinct large-scale network mechanisms to compensate for deficits in critical PI processing regions of the medial temporal lobe.

Unexpectedly, *APOE* ε4 carriers demonstrated superior PI estimation accuracy compared to non-carriers when local landmarks were available, despite higher insulin resistance. This effect was associated with enhanced segregation of the sensorimotor network. This finding is consistent with research indicating that spatially periodic grid cell firing and PI depend on error correction across visual, vestibular, and proprioceptive pathways [66-69]. While our participants did not benefit from proprioceptive cues due to the virtual task environment, studies have identified place, grid, boundary vector/border, head direction, and conjunctive cell activities in the primary somatosensory cortex of the rat, suggesting potential spatial tuning influenced by signals from the motor cortex via reciprocal connections [70]. Nevertheless, further research is required to determine if a similar specialization exists in the human somatosensory cortex.

It is important to emphasize that for the advantageous effect of *APOE* ɛ4 on PI to emerge in the presence of local landmarks, it was necessary to include trials with varying levels of difficulty, which is consistent with prior research [61]. This seemingly paradoxical beneficial effect of the ɛ4 isoform was previously noted in global cognition measures among individuals aged 45-55, yet no such association was found in those aged 56-75 [71]. Our earlier observation that younger individuals (<42 years) carrying a single copy of *APOE* ε4 did not show improved performance with spatial cues [6] aligns with meta-analyses conducted in this age group [72]. However, other studies conducted in young cohorts have reported beneficial effects of the ɛ4 allele on memory [73]. Future research should explore whether the observed effect in middle-aged adults reverses in individuals in their 70s and older, as suggested by studies examining other cognitive domains [71,74]. Confirmation of such findings would provide robust evidence supporting the antagonistic pleiotropy hypothesis of the *APOE* gene [75].

Carriers of the *APOE* ɛ4 genotype not only exhibit functional deficits of the entorhinal grid cell system [10] but also selective damage to hippocampal GABAergic interneurons [76]. This latter study has shown that GABAergic interneurons are particularly vulnerable to intracellularly *APOE* ɛ4, mediated through a tau-dependent mechanism, leading to their dysfunction and eventual degeneration. The loss of these interneurons results in hippocampal and cortical hyperexcitability and dysregulation of neural networks, potentially interfering with PI estimation. Aging exacerbates the detrimental impact of *APOE* ɛ4, increasing toxicity and further loss of GABAergic interneurons [76], which subsequently reduces brain system segregation [77]. The observed sensorimotor segregation benefit in *APOE* ɛ4 carriers when local landmarks are available may thus represent a compensatory response to dysfunction in the medial temporal lobe.

The large-scale brain system integration required by individuals with high insulin resistance to estimate PI regardless of spatial cues was both metabolically demanding and broadly impactful. Thus, it is plausible that vascular damage represents the primary mechanism driving this effect. This hypothesis is significantly bolstered by research linking brain insulin resistance in humans to reduced arterial blood flow and cerebral perfusion [78], increased risk of cerebrovascular disease [79], and decreased vascular insulin receptor concentrations observed in the parietal cortex of AD patients [80].

On the other hand, insulin has been recognized for its significant role in regulating neurovascular coupling [29], which helps explain how brain functional organization moderates the contribution of HOMA-IR to PI. Studies in mice lacking insulin receptors in astrocytes have shown central and peripheral insulin resistance [81]. This condition leads to a mismatch between brain blood flow and glucose uptake that worsens with age, likely due to impaired mitochondrial function in astrocytes and increased production of reactive oxygen species (ROS) [29].

Our findings suggest that varying levels of insulin resistance influence communication between cortical association systems differently, contributing to the variability in PI processes (see Table 6). Individuals with higher HOMA-IR values benefit from reduced segregation across both sensorimotor and associative networks. Those with intermediate HOMA-IR levels benefit from integrating the ventral attention network (VAN) and segregating the posterior multimodal network (PMN). Conversely, individuals with higher insulin sensitivity benefit from segregating sensorimotor networks and associative networks such as the default (DMN), dorsal attention (DAN), and cingulo-opercular network (CON). This shift in network dynamics with increasing insulin resistance suggests a reorganization of compensatory mechanisms. While the DMN and executive networks are critical for PI, as supported by their connections to medial temporal lobe structures specialized in spatial navigation [82], rising insulin resistance appears to shift reliance toward other networks also crucial for spatial navigation outside the medial temporal lobe, such as the PMN [51]. However, as insulin resistance worsens, greater network integration becomes necessary; while this may initially function as a compensatory mechanism, it may ultimately increase the vulnerability of these networks to age-related decline or neurodegenerative processes. These functional shifts could be explained by regional changes in neurovascular coupling, resulting from insulin depletion or reduced insulin receptor density in astrocytes. This progressive reorganization in functional network architecture may also account for the substantial variability in connectivity patterns observed among patients with type 2 diabetes [83]. While insulin resistance and obesity often co-occur in diabetes, approximately 20% of individuals with diabetes do not present with obesity-induced insulin resistance [84], which could further contribute to the heterogeneity in network responses and cognitive performance.

Another key finding of this study is that the moderating role of network segregation on the association between *APOE* genotype and PI was contingent upon GM integrity, whereas insulin resistance did not show a similar interaction. Specifically, the effect of the *APOE* ɛ4 appeared to be driven by variations in cortical thickness and intracortical myelin content. This influence was consistently observed across both cohorts, suggesting its independence from the metabolic profile of the sample and the specific characteristics of the navigation task. Given that *APOE* ɛ4 carriers exhibited regional changes in cortical thickness in both directions and a widespread loss of intracortical myelin content compared with non-carriers, one could hypothesize that the contribution of system segregation to the influence of *APOE* ɛ4 on PI might be attributed to the impact of GM atrophy on FC and/or system segregation. Consistent with this hypothesis, there is evidence linking age-related changes in resting-state FC to variability in cortical thickness [35] and intracortical myelin [49,37], and age-related changes in brain system segregation to increased cortical atrophy [85]. However, in our study, these associations either did not reach statistical significance, or if they did, the effect size was deemed negligible. The fact that GM did not affect how brain functional organization modulates the impact of insulin resistance on PI performance reinforces the notion that *APOE* ε4 carriers and individuals with insulin resistance rely on different neural pathways to mitigate deficits in key regions responsible for PI processes.

### Impact of cohort-specific metabolic profiles and GM integrity on network dynamics and PI performance

This study revealed significant differences in metabolic profiles, GM integrity and network segregation between the S_longAG_ and S_shortAG_ cohorts, which affected their PI abilities. The S_longAG_ exhibited a more adverse metabolic profile, characterized by higher HOMA-IR values, along with greater dyslipidemia and overall obesity prevalence. Chronic inflammation and insulin resistance in this cohort likely impaired the function of associative networks [86-88], leading to reduced overall network segregation and diminished functional specialization of specific networks. This impairment may hinder cognitive processes essential for PI, particularly within the CON and FPN, both of which are critical for managing attention, task execution, spatial awareness, and decision-making [89,90]. Consistent with this hypothesis, recent evidence has linked late-life cognitive decline to increased phosphorylation of the serine/threonine kinase (AKT)—a marker of brain insulin resistance—in the prefrontal cortex [91].

Conversely, the S_shortAG_ cohort, characterized by a higher prevalence of central obesity and hypertension, may experience not only neuroinflammation but also altered cerebral perfusion, both of which influence network dynamics and performance in PI. Although studies, such as those by Bu et al. [92], have demonstrated that hypertension is associated with reduced network interaction, suggesting disturbed neurovascular coupling, our findings revealed that individuals in this cohort can still benefit from enhanced connectivity among less segregated networks, such as the VAN, CON, and FPN, during PI. This paradox may be explained by the fact that, while hypertension can impair overall network integration, the specific functional demands of the navigation task may rely on greater interaction among certain networks to compensate for the deficits. Furthermore, the greater GM integrity observed in the S_shortAG_ cohort likely supports this connectivity, allowing them to maintain better overall network health despite their cardiovascular challenges. This contrasts with the S_longAG_ cohort, which shows structural adaptations indicative of a negative metabolic profile. In this cohort, insulin resistance and chronic inflammation could act as synergistic factors that exacerbate the negative metabolic profile [93], further impairing spatial navigation, as indicated by research linking these conditions to neuroinflammation and neuronal loss [94].

The differences between cohorts highlight how network segregation interacts with cognitive performance in spatial navigation, particularly concerning metabolic health indicators such as HOMA-IR and genetic factors like the *APOE* ε4 allele. While both cohorts benefited from the integration of sensorimotor and associative networks for PI estimation, the specific networks that contributed to these processes varied between cohorts potentially due to differences in HOMA-IR levels and other metabolic factors. In individuals with higher insulin resistance, who were part of the S_longAG_ cohort, the integration of visual and associative networks supported more accurate PI, suggesting that broader network connectivity helps compensate for metabolic impairments. In contrast, individuals with intermediate HOMA-IR values, who were primarily in the S_shortAG_ cohort, also benefited from the integration of different sensorimotor and associative networks, but crucially showed better PI performance when the PMN, a network specifically implicated in spatial navigation [51], was more segregated. This suggests that while network integration aids PI across both cohorts, individuals with milder metabolic disturbances may rely more on the specialized functioning of spatial navigation networks like the PMN.

The influence of the *APOE* ε4 allele adds complexity to the results. In the S_longAG_ cohort, ε4 carriers benefited from the segregation of the VN2, which may help mitigate the cognitive impairments associated with their elevated IR and overall metabolic challenges. Conversely, ε4 carriers in the S_shortAG_ cohort showed enhanced PI through the integration of the VAN, CON, and FPN, suggesting that this network connectivity may address their specific cardiovascular risks. Overall, these findings emphasize the importance of network dynamics in cognitive function, illustrating how different metabolic conditions shape the reliance on network integration and segregation to support effective PI.

In summary, the interplay between metabolic health, network segregation, and cognitive performance highlights the need to consider individual differences in cardio-metabolic profiles when examining brain network interactions. Targeted interventions aimed at enhancing network connectivity may be beneficial, particularly for populations exhibiting metabolic impairments, stressing the importance of personalized approaches in cognitive health strategies.

### Limitations

The present study has several limitations that warrant consideration. First, the relatively small sample sizes and wide age range may have limited the statistical power, particularly for genetic analyses, and introduced variability related to age. While age was controlled as a covariate, larger and more homogeneous samples would likely yield more robust results and enhance the detection of significant effects. Despite these limitations, the consistency of primary findings across both cohorts, which performed tasks with varying cognitive demands, supports the external validity of our results.

Second, the cross-sectional design limits our ability to establish causality. It remains unclear whether changes in network segregation lead to cognitive impairments, or whether pre-existing cognitive deficits alter brain network configurations. Longitudinal studies are required to better elucidate these causal pathways.

Finally, the study does not provide direct evidence from clinical populations with neurodegenerative conditions, and the generalizability of the findings may be limited by demographic factors such as age, health status, and lifestyle. Future research should investigate whether the observed associations hold in individuals with AD or other forms of dementia, as well as in more diverse clinical populations, to improve the broader applicability of the results.

### Conclusion

The results of the present study suggest that the *APOE* ɛ4 genotype and insulin resistance influence PI performance through different large-scale network mechanisms. Insulin resistance is linked to the activation of metabolically costly mechanisms, which may increase vulnerability to aging and neurodegeneration. With the rising prevalence of obesity and type 2 diabetes, particularly among younger populations, the risk of AD is significantly heightened. While further controlled intervention studies are necessary to confirm whether enhancing insulin sensitivity in the brain of individuals with cardiovascular and neurodegeneration risk factors truly benefits metabolism and cognition, current evidence is promising [95,96]. Overall, these findings highlight the critical importance for healthcare systems to effectively manage insulin resistance in midlife as a preventing strategy, supporting the World Health Organization’s goal of enhancing the functional capacity of older adults, especially those at heightened risk of AD.

## Supplementary material

The Supplementary data can be found online at: www.aginganddisease.org/EN/10.14336/AD.2023.0975.
