# Supplementary material for: APOE ɛ4 and Insulin Resistance Influence Path-Integration-Based Navigation through Distinct Large-Scale Network Mechanisms
Source: Aging Dis. 2024 Nov 18;16(5):3154–79. doi: 10.14336/AD.2024.0975 (PMC12339104; doi:10.14336/AD.2024.0975)
Supplement: Supplementary file 1 [file AD-16-5-3154-s.pdf]

SUPPLEMENTARY DATA

APOE ε4 and Insulin Resistance Influence Path-Integration-Based Navigation through Distinct Large-Scale Network Mechanisms

Karel M. Lopez-Vilaret, Marina Fernandez-Alvarez, Anne Bierbrauer, Nikolai Axmacher, Jose L. Cantero, Mercedes Atienza

© 2024. Lopez-Vilaret KM. et al. Published online at http://www.aginganddisease.org/EN/10.14336/AD.2024.0975

SUPPLEMENTARY DATA

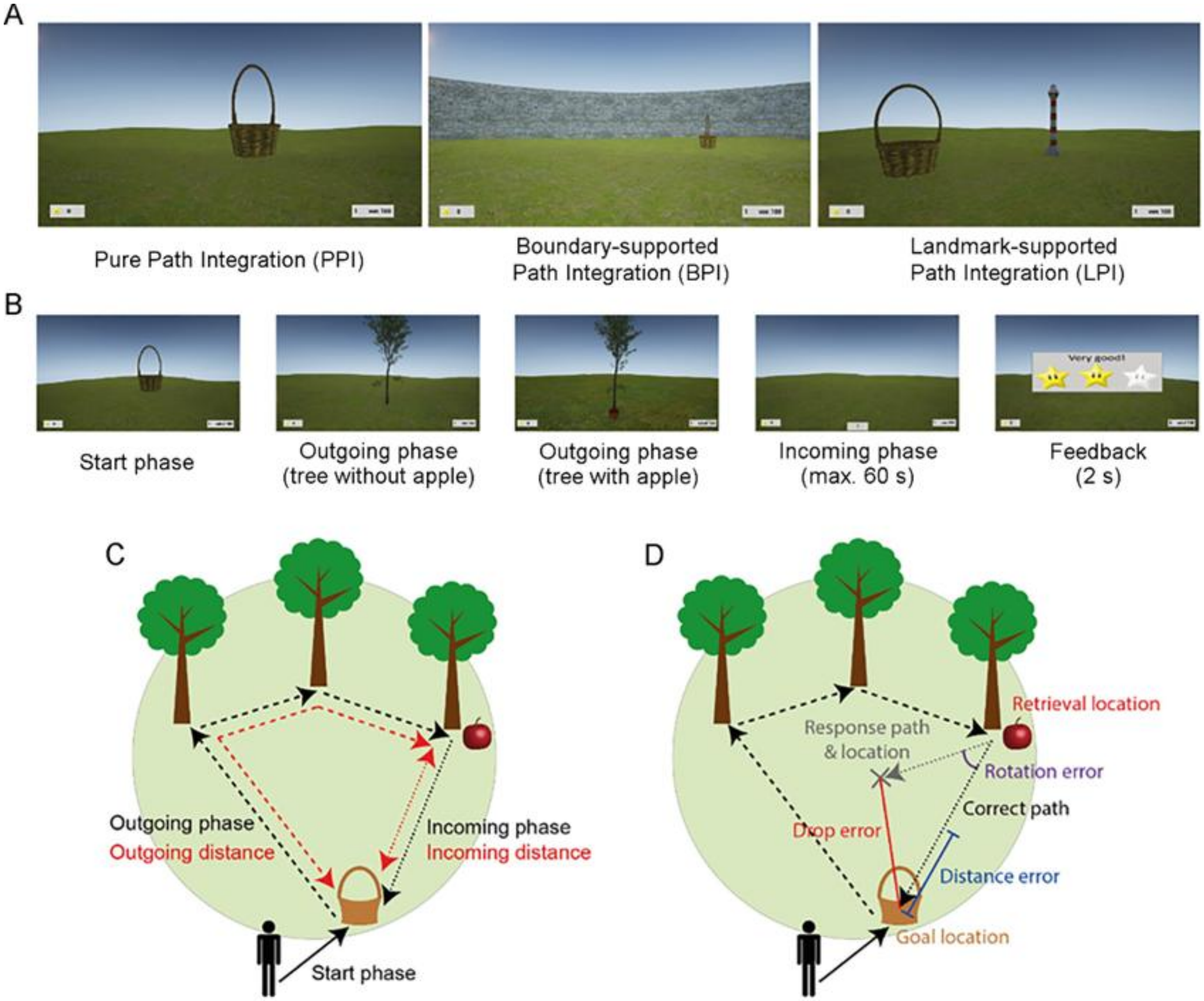

Supplementary Figure 1. Illustration of the Apple Game task. (A) The task comprised three subtasks: the PPI subtask without any supportive cue; the BPI subtask with a circular boundary; and the LPI subtask with an intra- maze landmark (lighthouse) close to the center of the environment. (B) In each trial, participants collected a basket (start phase) and tried to remember its location (goal location). After navigating towards a variable number of trees (0-4; outgoing phase), which disappeared after having been reached, participants had to find their way back to the goal location (incoming phase). Finally, they received feedback via different numbers of stars, depending on response accuracy. (C) Outgoing distance refers to the cumulated distance during the outgoing phase; incoming distance refers to the Euclidean distance between retrieval location (tree with apple) and goal location (basket). (D) PI performance was assessed as the distance between the correct goal location and the response location (drop error). Modified from [6].

© 2024. Lopez-Vilaret KM. et al. Published online at http://www.aginganddisease.org/EN/10.14336/AD.2024.0975

SUPPLEMENTARY DATA

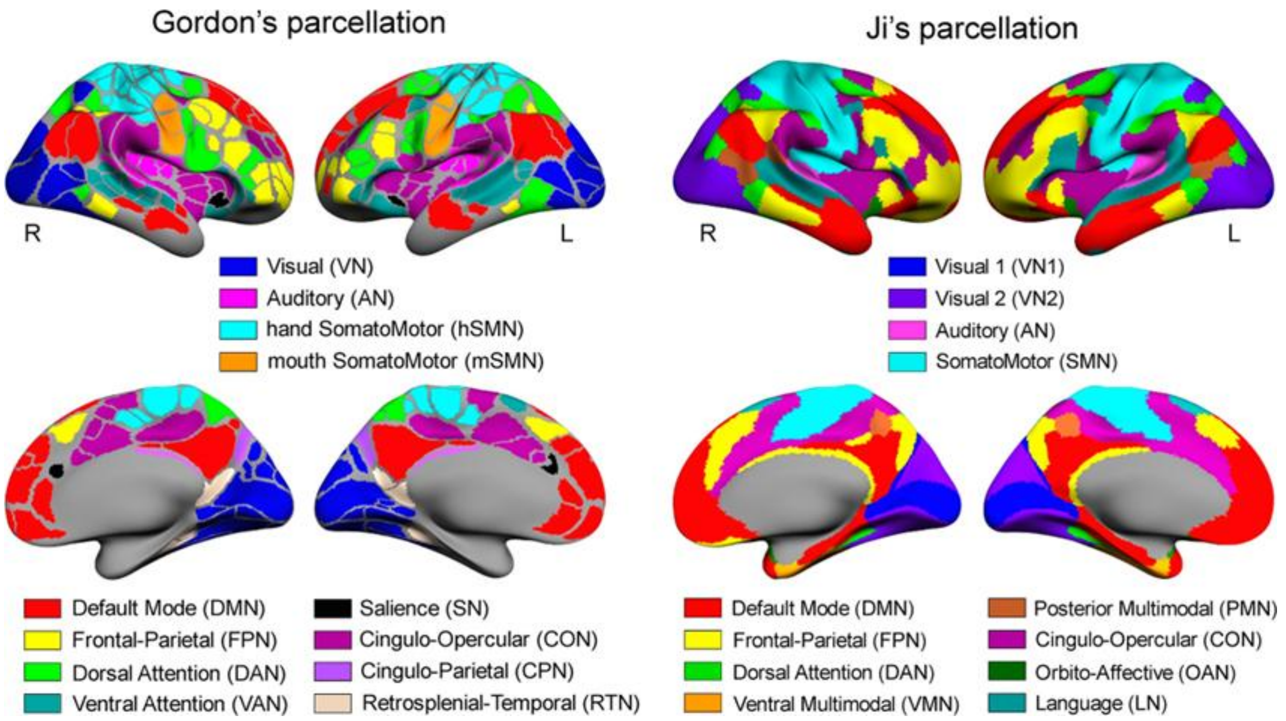

Supplementary Figure 2. Parcellation schemes and functional networks. Cortical parcels and functional networks included in the Gordon [49] (left panel) and Ji [51] parcellation scheme (right panel). R: right; L: left.

Supplementary Table 1. Number of parcels per brain network in the parcellation scheme of Gordon [49] and Ji [51].

Gordon	LH / RH	Ji	LH / RH
VN	18 / 21	VN1	3 / 3
AN	12 / 12	VN2	27 / 27
hSMN	18 / 20	AN	8 / 7
mSMN	4 / 4	SMN	19 / 20
DMN	20 / 21	DMN	40 / 37
FPN	9 / 15	FPN	22 / 28
DAN	19 / 13	DAN	12 / 11
VAN	11 / 12	VMN	2 / 2
SN	2 / 2	PMN	3 / 4
CON	20 / 20	CON	27 / 29
CPN	3 / 2	OAN	3 / 3
RTN	4 / 4	LN	14 / 9
None	21 / 26		

© 2024. Lopez-Vilaret KM. et al. Published online at http://www.aginganddisease.org/EN/10.14336/AD.2024.0975
